# Optimisation of microalgal cultivation via nutrient-enhanced strategies: the biorefinery paradigm

**DOI:** 10.1186/s13068-021-01912-2

**Published:** 2021-03-12

**Authors:** Gonzalo M. Figueroa-Torres, Jon K. Pittman, Constantinos Theodoropoulos

**Affiliations:** 1 Department of Chemical Engineering and Analytical Science, Biochemical and Bioprocess Engineering Group, The University of Manchester, Manchester, M13 9PL UK; 2 Department of Earth and Environmental Sciences, The University of Manchester, Manchester, M13 9PL UK

**Keywords:** Modelling, Biofuels, Starch, Lipids, Biorefinery, Microalgae, *Chlamydomonas*, Optimisation, Nutrient limitation, Mixotrophy

## Abstract

**Background:**

The production of microalgal biofuels, despite their sustainable and renowned potential, is not yet cost-effective compared to current conventional fuel technologies. However, the biorefinery concept increases the prospects of microalgal biomass as an economically viable feedstock suitable for the co-production of multiple biofuels along with value-added chemicals. To integrate biofuels production within the framework of a microalgae biorefinery, it is not only necessary to exploit multi-product platforms, but also to identify optimal microalgal cultivation strategies maximising the microalgal metabolites from which biofuels are obtained: starch and lipids. Whilst nutrient limitation is widely known for increasing starch and lipid formation, this cultivation strategy can greatly reduce microalgal growth. This work presents an optimisation framework combining predictive modelling and experimental methodologies to effectively simulate and predict microalgal growth dynamics and identify optimal cultivation strategies.

**Results:**

Microalgal cultivation strategies for maximised starch and lipid formation were successfully established by developing a multi-parametric kinetic model suitable for the prediction of mixotrophic microalgal growth dynamics co-limited by nitrogen and phosphorus. The model’s high predictive capacity was experimentally validated against various datasets obtained from laboratory-scale cultures of *Chlamydomonas reinhardtii* CCAP 11/32C subject to different initial nutrient regimes. The identified model-based optimal cultivation strategies were further validated experimentally and yielded significant increases in starch (+ 270%) and lipid (+ 74%) production against a non-optimised strategy.

**Conclusions:**

The optimised microalgal cultivation scenarios for maximised starch and lipids, as identified by the kinetic model presented here, highlight the benefits of exploiting modelling frameworks as optimisation tools that facilitate the development and commercialisation of microalgae-to-fuel technologies.

**Supplementary Information:**

The online version contains supplementary material available at 10.1186/s13068-021-01912-2.

## Background

The commercialisation of biofuels, which are potentially promising and sustainable substitutes for fossil-based fuels, has been severely restricted to current feedstock technologies which largely rely upon the use of traditional food-based or lignocellulosic biomass [1–4]. The on-going search for sustainable and renewable feedstock alternatives has led to the recognition of microalgae as a promising long-term feedstock (known as third-generation) capable of meeting global biofuel demands [1, 3, 5–7]. The potential of microalgae is highlighted by the typical fast growth rate of many strains, leading to high biomass production, and the ability to accumulate carbohydrate (mainly in the form of starch) and lipids, precursor molecules for sugar-based and oil-based biofuels [8, 9]. Since their cellular composition also includes other industrially important biomolecules (e.g. proteins, pigments, vitamins, and other bioactive compounds) [5, 10], microalgae are now positioned as a viable biomass feedstock for biorefineries [5, 11, 12].

**Fig. 1:**
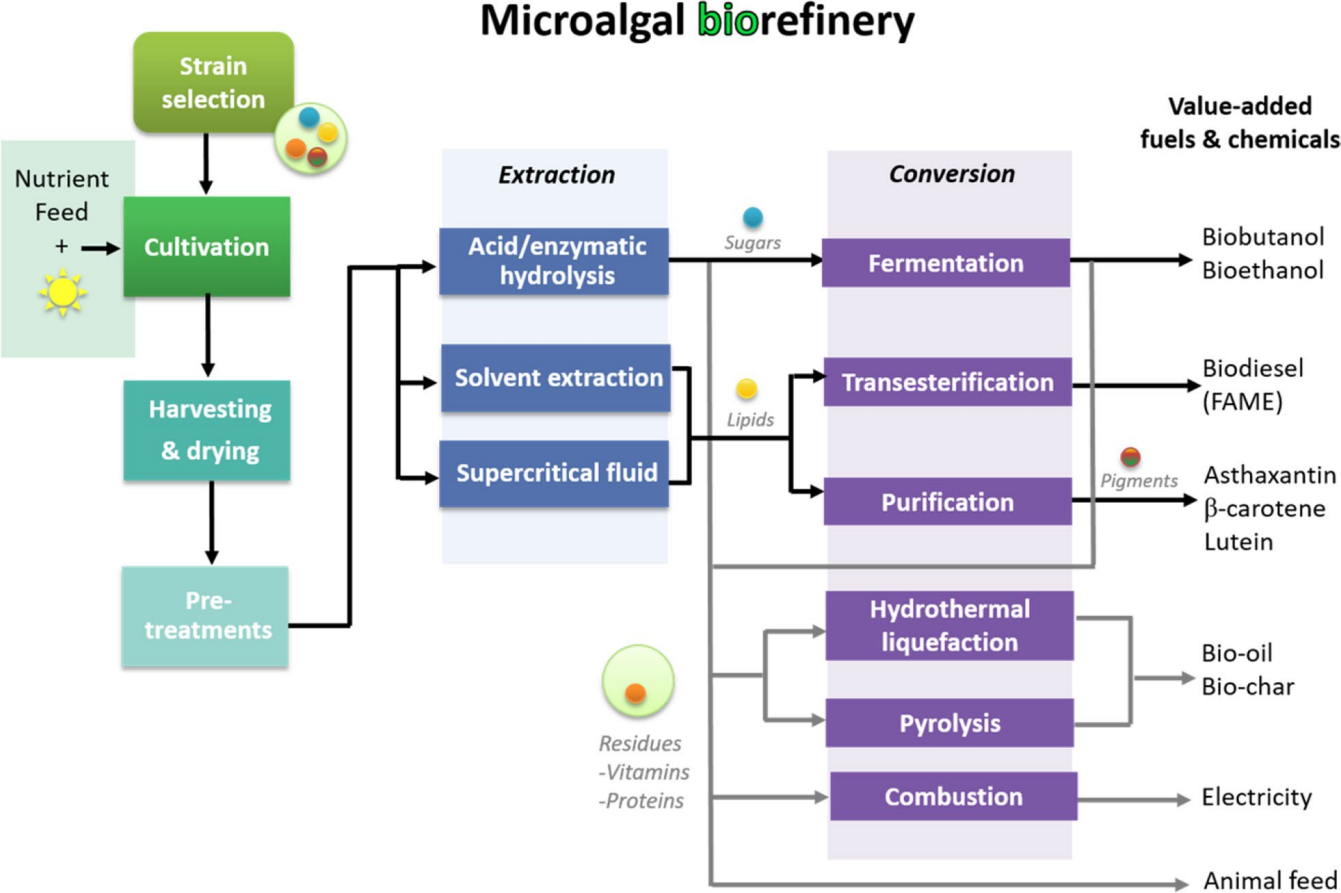
Schematic representation of a microalgal biorefinery for the co-production of biofuels and value-added chemicals

Through the full exploitation of the rich cellular composition of microalgae, microalgal biorefineries offer a profitable and competitive approach for the co-production of biofuels along with other high value-added chemicals [12, 13]. Figure 1 shows some of the conversion routes that could be implemented within a biorefinery framework to obtain biofuels and other commercially important products. The implementation of such a framework can help increase energy efficiency and process profitability by optimally integrating all possible bioprocessing routes along with waste re-valorisation scenarios [5, 11].

The prospects of microalgae biorefineries are encouraging [13–15], and available technoeconomic analyses show that integrating biofuels conversion routes with other high-value chemicals and/or energy generation systems (e.g. hydrogen, methane) in an integrated biorefinery concept is more economically attractive [16–18]. Technoeconomic studies, however, should be interpreted cautiously due to the often high number of assumptions and degrees of freedom typically used to obtain economic projections. Indeed, despite the promising advantages of integrating biofuels production within a biorefinery network, microalgal biofuel technologies are not yet sufficiently developed to be competitive on their own [19, 20]. This represents a major drawback that currently prevents microalgal biofuels from being commercialised.

A prevalent challenge of microalgae technologies for biofuels production purposes is the need to identify cultivation strategies that generate mass-scale microalgal cultures rich in starch and lipids, i.e. the biofuel precursors [21–23]. In this regard, it has been widely demonstrated that the cultivation environment can be artificially manipulated to induce starch and lipid accumulation [24, 25]. Nutrient-stressed cultivation strategies (e.g. nitrogen or phosphorous limitation), in particular, have been established as a simple, cost-effective strategy for enhanced starch and lipid formation [24, 26–30]. Nevertheless, nutrient limitation often drastically reduces microalgal growth, which in consequence reduces total starch and lipid productivities [22, 30, 31].

Mixotrophically grown strains (i.e. those that assimilate organic carbon sources in addition to inorganic carbon dioxide) generally attain higher growth rates than typical phototrophic strains (i.e. those that rely solely on inorganic carbon dioxide) [31–33], and thus have the potential of withstanding any adverse effects caused by nutrient limitation. Implementing starch/lipid-enhancing strategies, therefore, relies on the challenging optimisation of microalgae’s nutritional requirements (e.g. carbon, nitrogen, phosphorous, etc.) in such a way that the trade-off between microalgal growth and starch and lipid formation is effectively balanced. Predictive models of microalgal growth are therefore essential tools towards identifying optimal cultivation strategies [34].

Here, we present an experimentally validated predictive model for nutrient-limited, mixotrophic microalgal growth that reflects carbon assimilation and carbon partitioning between starch and lipid reserves. The model was exploited to identify nutrient-enhanced microalgal cultivation strategies, which yielded a significant increase in starch (+ 270%) and lipid (+ 74%) production compared to a non-optimised scenario. The optimisation framework that we show here (combining both modelling and experimental methodologies) can thus be applied for the systematic identification of optimal cultivation strategies, increasing the likelihood of establishing competitive biofuel-oriented microalgal biorefineries.

## Results

### Evaluating microalgal responses to media composition

In order to build a predictive model capable of portraying nutrient-limited mixotrophic dynamics, we first quantified the effects of initial nutrient availability on microalgal growth and starch and lipid accumulation. To do so, laboratory-scale cultivation experiments were carried out with the model green microalgae *Chlamydomonas reinhardtii* subject to different initial concentrations (Additional file 1: Table S1) of nitrogen, phosphorus, and acetic acid (as an organic carbon source) until the stationary phase was achieved (8 days). The concentrations of nitrogen, phosphorus, and acetic acid in a standard pH-buffered artificial growth medium (TAP medium) commonly used for laboratory cultivation of *C. reinhardtii* [35], were used as the reference case against which all other nutrient modified cultures were statistically compared (“Methods”). The results of the biomass, starch and lipid responses are summarised in Table 1.

**Table 1:** Biomass, starch, and lipid concentrations in *C. reinhardtii* at *t* = 192 h

Treatment	Biomass gC L^−1^		Starch gC L^−1^		Starch %		Lipids gC L^−1^		Lipid %	
(TAP)	0.318	–	0.0179	–	5.6%		0.0448	*–*	14.1%	
(Low P: Low N)	0.247	*	0.0414	***	16.8%	***	0.0436		17.7%	
(Low N)	0.281		0.0473	***	16.8%	***	0.0596	*	21.2%	***
(Med N)	0.305		0.0309	***	10.1%	*	0.0566		18.6%	*
(Low P)	0.267		0.0302	***	11.3%	**	0.0415		15.6%	
(Med P)	0.294		0.0208		7.1%		0.0419		14.3%	
(Low A)	0.259		0.0128		4.9%		0.0383		14.8%	
(High A)	0.390	*	0.0220		5.6%		0.0666	***	17.1%	
(High A+)	0.414	**	0.0380	***	9.2%		0.0758	***	18.3%	
(High A: Low N−)	0.234	*	0.0536	***	22.9%	***	0.0479		20.5%	**
(High A: Low P)	0.372		0.0304	***	8.2%		0.0620	**	16.7%	
(High N++)	0.168	***	0.0141		8.4%		0.0242	***	14.4%	
(High P++)	0.294		0.0155		5.3%		0.0431		14.7%	
(High A++)	0.294		0.0178		6.1%		0.0421		14.3%	

Asterisks (*) denote significant differences (*p* < 0.05*, 0.01**, 0.001***) with respect to (TAP), as per one-way ANOVA. Data are the mean of two independent biological replicates

Unmodified (TAP) medium composition yielded a biomass concentration of 0.318 gC L^−1^, consisting of 5.6% starch and 14.1% lipid. When compared to (TAP), all nutrient-limited conditions caused minor reduction in biomass. However, the only statistically significant reduction was observed in the culture grown under simultaneous phosphorus and nitrogen limitation (Low P: Low N) conditions (*p* = 0.048, one-way ANOVA), where biomass concentration dropped − 22% with respect to (TAP). In line with previous observations [26], nitrogen limitation (Low N) significantly increased both starch and lipid contents up to 16.8% and 21.2%, respectively (Table 1). In contrast, phosphorus limitation (Low P), significantly induced starch accumulation up to 11.3% (*p* = 0.006) but lipid accumulation was not significantly different under any of the phosphorus-limited scenarios. This included the (Low P: Low N) conditions, where only starch concentration increased significantly (*p* < 0.001) with respect to (TAP). Accumulation of starch rather than lipid molecules during phosphorus limitation can be explained by starch synthesis being the preferred product of carbon assimilation in *C. reinhardtii* [36], or by the phosphate-associated inhibition of ADP-glucose pyrophosphorylase which regulates starch synthetic pathways [37, 38].

Increasing acetic acid concentration significantly increased biomass concentrations up to 23% in (High A) (*p* = 0.043) and 30% in (High A+) (*p* = 0.009) conditions (Table 1). Acetic acid-associated induced growth in *C. reinhardtii* has been previously described as a consequence of enhanced mixotrophic growing conditions [31, 32]. High acetic acid concentration subject to low phosphorus (High A: Low P) similarly supported higher biomass with respect to (TAP), whereas biomass decreased significantly (*p* = 0.026) in combination with low nitrogen (High A: Low N), which indicates the more important role that nitrogen plays in sustaining microalgal growth. With respect to (TAP), the high acetic acid cultures yielded an increase in starch and lipid concentrations, and correspondingly, an increase in contents of up to 9.2% starch and 18.3% lipids, as observed in the (High A+) treatment. The increase in starch and lipid contents, however, was not statistically significant and thus mainly associated to the higher biomass supported by the internal acetate boost. An exception was the (High A: Low N−) culture, which accumulated significantly more starch (22.9%, *p* < 0.001) and lipids (20.5%, *p* = 0.002) than (TAP) due to the combined effect of the acetate boost with nitrogen stress. This highlights the greater effect of nitrogen over phosphorus limitation in starch and lipid accumulation. Increased lipid concentrations during acetic acid-enhanced cultivation were similarly reported by Bekirogullari et al. [31, 39], while considerably higher accumulation of lipid has been observed in the starch-less (*sta6*) mutant strain when subject to an acetate boost and nitrogen limitation [40, 41].

Extreme high nutrient concentrations [i.e. (High N++), (High P++), and (High A++)] inhibited biomass growth in all cases, but significantly so in the (High N++) treatment (*p* < 0.001) which caused a biomass drop of − 47%. With regard to starch and lipids, these treatments yielded only small increases of up to 8.4% starch [in (High N++)] and 14.7% lipids [in (High P++)], and were deemed not significant as per the statistical analysis (Table 1). Therefore, these strategies are inappropriate for large-scale microalgal cultivation. Exploring the full effect of nutrient concentrations on microalgal dynamics is of vital importance to select optimal nutritional composition, but this evaluation requires costly and time-consuming experimental analyses. Therefore, we employed the data collected here to construct, and subsequently validate, a predictive kinetic model for microalgal growth.

### Building a predictive model for microalgal growth

**Fig. 2:**
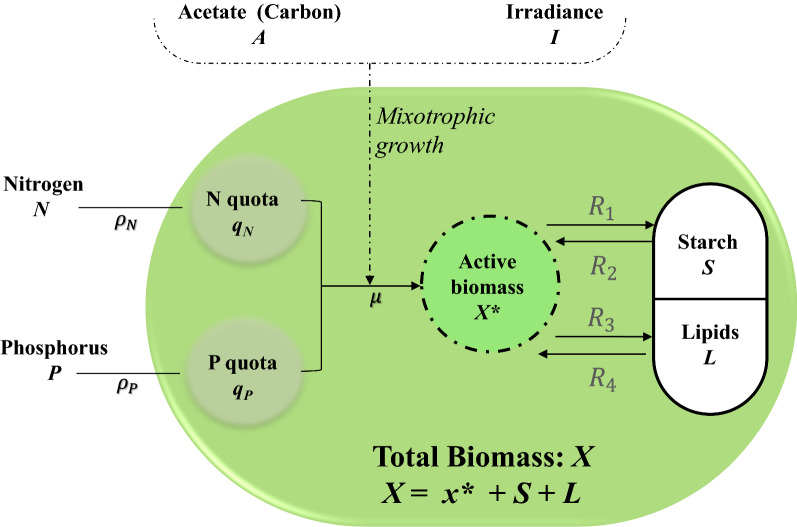
Schematic representation of the cellular compartments and flows used in the kinetic model.* μ*, specific growth rate; ${{\varvec{\rho}}}_{{\varvec{N}}}$, nitrogen uptake rate; ${{\varvec{\rho}}}_{{\varvec{P}}}$, nitrogen uptake rate; ${{\varvec{R}}}_{1}$*,* starch synthetic rate; ${{\varvec{R}}}_{3}$, lipid synthetic rate; ${{\varvec{R}}}_{2}$, starch degradation rate; ${{\varvec{R}}}_{4}$, lipid degradation rate

We previously developed a kinetic model for mixotrophic microalgal growth, alongside starch and lipid formation, as a function of initial nitrogen and organic carbon (acetic acid) concentrations [42]. Here, we present a model with markedly improved predictive capabilities by: (i) taking into account the effects of phosphorus concentration on the algal cultivation dynamics, (ii) incorporating the average light intensity received by the microalgal culture, and (iii) improving the starch and lipid formation rate equations. The model state variables are: total biomass *X* (gC L^−1^), starch *S* (gC L^−1^), lipids *L* (gC L^−1^), active biomass x* (gC L^−1^), nitrogen *N* (gN L^−1^), nitrogen quota *q*_*N*_ (gN gC_x_^−1^), phosphorus *P* (gPO_4_ L^−1^), phosphorus quota *q*_*P*_ (gPO4 gC^−1^), and acetic acid *A* (gC_A_ L^−1^). Total biomass is assumed to be the sum of active biomass, starch, and lipids (Fig. 2). The model governing equations are explained below.

#### Specific growth rate

The specific growth rate, $\mu$ (h^−1^), which describes how cells grow over a period of times is expressed by a quadruple-factor function incorporating the effects of nitrogen, phosphorus, acetic acid, and the light received by the culture:

1 $$\mu = \overline{\mu }_{{M,{\text{max}}}} \left( {A,\overline{I}} \right) \cdot {\text{min}}\left[ {\mu_{N} \left( {q_{N} } \right),\mu_{P} \left( {q_{P} } \right)} \right].$$

The nitrogen-limited, ${\mu }_{N}$, and phosphorus-limited, ${\mu }_{P}$, growth rates are subject to a minimum law and are each expressed as Droop functions [43] of the nitrogen quota, ${q}_{N}$, and the phosphorus quota, ${q}_{P}$, respectively:

2 $$\mu_{N} \left( {q_{N} } \right) = 1 - \frac{{q_{N,0} }}{{q_{N} }}; \mu_{P} \left( {q_{P} } \right) = 1 - \frac{{q_{P,0} }}{{q_{P} }}.$$

Here, ${q}_{N,0}$ (gN gC^−1^) and ${q}_{P,0}$ (gPO_4_ g C^−1^) are the minimum nitrogen and phosphorus quotas required to sustain growth, respectively. The maximum mixotrophic specific growth rate, ${\stackrel{-}{\mu }}_{M,\mathrm{max}}(A,\stackrel{-}{I})$, is regulated by the acetate-driven heterotrophic growth rate, ${\mu }_{H}$, and the light-driven phototrophic growth rate, ${\mu }_{I}$, both described by an Andrews function [44] to portray substrate-inhibition and photoinhibition, respectively:

3 $$\overline{\mu }_{{M,{\text{max}}}} \left( {A,\overline{I}} \right) = \mu_{{{\text{max}}}} \cdot \left[ {w_{H} \cdot \mu_{H} \left( A \right) + w_{I} \cdot \mu_{I} \left( {\overline{I}} \right)} \right],$$

4 $$\mu_{H} \left( A \right) = \frac{A}{{A + K_{S,A} + A^{2} /K_{i,A} }}; \mu_{I} \left( {\overline{I}} \right) = \frac{{\overline{I}}}{{\overline{I} + K_{S,I} + \overline{I}^{2} /K_{i,I} }}.$$

Here, ${\mu }_{\mathrm{max}}$ (h^−1^) is the maximum mixotrophic specific growth rate, ${K}_{S,A}$ and ${K}_{i,A}$ (gC L^−1^) are the acetate-associated half-saturation and inhibition constants, respectively, and ${K}_{S,I}$ and ${K}_{i,L}$ (μ_mol_ m^−2^ s^−1^) are the light-associated half-saturation and inhibition constants, respectively; ${w}_{H}$ and ${w}_{I}$ are weighing functions controlling the magnitude of the heterotrophic and phototrophic growth rates, respectively.

Microalgal productivity is dependent on the total amount of photosynthetically active radiation (in the range of 400–700 nm) received by microalgal cells [45]. However, even though light might be supplied at a constant intensity, the amount of light received is affected by the cell absorption efficiency as well as by scattering, reflection, and refraction processes [46]. In addition, different wavelengths affect growth rates among microalgae species [47, 48]. Whilst an accurate representation of the light received by microalgal cultures should account for the above phenomena, a more common yet simple modelling approach relies on estimating the light, *I* (μ_mol_ m^−2^ s^−1^), at a given culture depth,* z* (m), by means of the Beer-Lambert law, which assumes that light decreases exponentially with increasing biomass growth. Since cells can receive more light at the culture surface than at the bottom, here we use a slightly more accurate representation of the light throughout the vessel by computing an average light intensity, $\stackrel{-}{I}$, between the surface ($z=0$), and its depth ($z=L$), so that [49]:

5 $$\overline{I} = \frac{{I_{o} }}{L}\mathop \smallint \limits_{0}^{L} e^{ - \sigma \cdot X \cdot z} \cdot {\text{d}}z = \frac{{I_{0} }}{\lambda } \cdot \left( {1 - e^{ - \lambda } } \right),$$

where $\sigma$ (L gC^−1^ m^−1^) is the light attenuation coefficient, and $\lambda = \sigma \cdot X \cdot L$ is the optical depth. It is worth noting that the optical depth can be further improved by considering that light attenuation depends not only on biomass growth, but also on the concentration of chlorophyll and other pigments [50]. The day:light cycle is additionally known to have an effect on biomass growth and in starch and lipid formation. In *Chlamydomonas*, for example, accumulation of starch and lipids (TAG) was observed to be dependent on whether cells are grown in the light or in the dark [36]. More interestingly, starch accumulation has been reported to be controlled by a circadian clock (as opposed to simple *lights-on lights-off* periods) which is discontinued following nitrogen starvation [51]. The model presented here aims to portray the macroscopic dynamics of microalgae as a function of nutrient composition and therefore does not consider the photoperiod, however the simulation of the specific light–dark cycle can help in the understanding of more complex metabolic pathways occurring in microalgae between day and night.

#### Nitrogen and phosphorus uptake rates

Microalgae models commonly employ simple saturation-type kinetics to simulate nutrient consumption [43, 52, 53]. In this model, however, the nitrogen uptake rate employs inhibition-type kinetics dependent on nitrogen to account for the growth inhibition observed at a high nitrogen concentration [i.e. (High N++), Table 1], but also dependent on acetic acid to account for the reduction of nitrogen consumption observed at a high acetic acid concentration [i.e. (High A++), Additional file 1, Table S3]. The nitrogen uptake rate, ${\rho }_{N}$ (gN gC^−1^ h^−1^), is therefore expressed as follows:

6 $$\begin{aligned} \rho_{N} =& \overline{\rho }_{{N,{\text{max}}}} \left( {N_{0} ,X} \right) \cdot \frac{N}{{N + k_{s,N} + N^{2} /k_{i,N} }}\\ & \cdot \frac{A}{{A + k_{s,A:N} + A^{2} /k_{i,A:N} }} \cdot f\left( {q_{P} } \right). \end{aligned}$$

Here, ${k}_{s,N}$ and ${k}_{i,N}$ (gN L^−1^) are nitrogen-associated half-saturation and inhibition constants, respectively, and ${k}_{s,A:N}$ and ${k}_{i,A:N}$ (gC L^−1^) are acetate-associated half-saturation and inhibition constants, respectively.

In Eq. (6), ${\stackrel{-}{\rho }}_{N,\mathrm{max}}({N}_{o},X)$ is the maximum nitrogen uptake rate, which accounts for the luxury uptake of nitrogen of microalgal cells (i.e. a phenomenon where the uptake of nutrient is fast immediately after inoculation). Given that the extent of luxury uptake was thought to be dependent on the nutrient concentration of the “fresh” medium and the cell density [54], the maximum nitrogen uptake rate is regulated by the initial nitrogen medium concentration, ${N}_{0}$, and the biomass concentration, $X$, as:

7 $$\overline{\rho }_{{N,{\text{max}}}} \left( {N_{0} ,X} \right) = \rho_{{N,{\text{max}}}} \cdot \frac{{N_{o}^{n} }}{{N_{o}^{n} + K_{*}^{n} }} \cdot e^{{ - \phi_{N} \cdot X}} .$$

Here ${\rho }_{N,max}$ (gN gC^−1^ h^−1^) is the maximum nitrogen uptake rate, ${\phi }_{N}$ is an uptake regulation coefficient (L gC^−1^), $n$ is a shape-controlling parameter, and ${K}_{*}$ is a saturation constant (gN L^−1^). In Eq. (7), the effect of the initial nitrogen concentration is described using saturation-type kinetics, and the effect of biomass is expressed by an exponential term indicating that the uptake of nitrogen decreases exponentially with increasing biomass concentration.

**Fig. 3:**
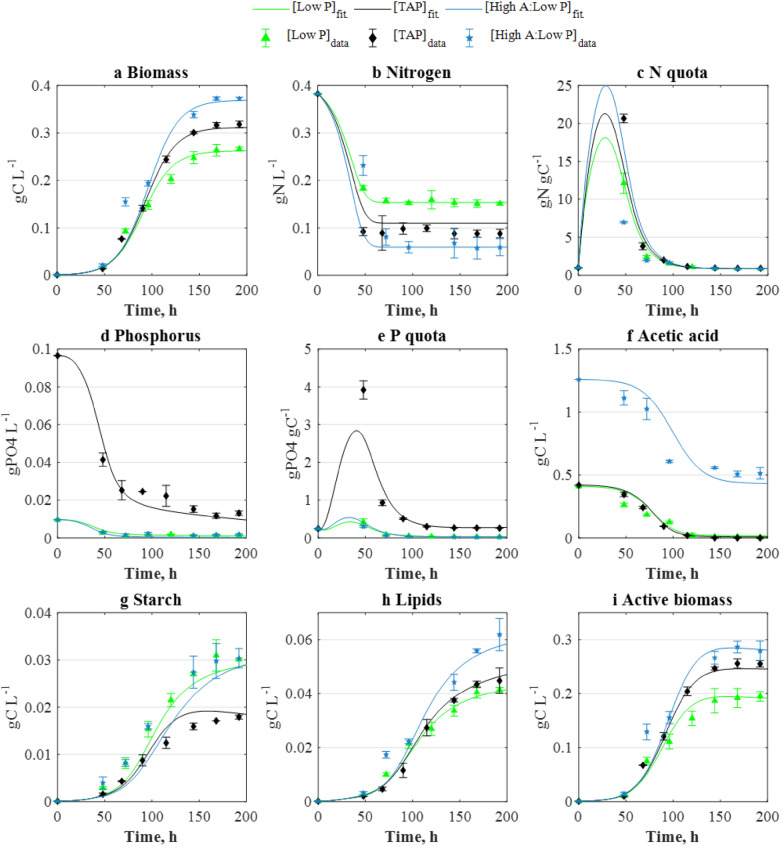
Comparison between the experimental (points) concentration–time profiles and the model fittings resulting from parameter estimation. (TAP): *N*_*0*_ = 0.382 gN L^−1^, *P*_*0*_ = 0.096 gPO_4_ L^−1^, *A*_*0*_ = 0.42 gC L^−1^, [Low P]: 0.382 gN L^−1^, 0.0096 gPO_4_ L^−1^, 0.42 gC L^−1^, and (High A: Low P): 0.382 gN L^−1^, 0.0096 gPO_4_ L^−1^, 1.26 gC L^−1^. Data and error bars represent the mean and range (min/max values), respectively, of two independent experimental replicates

**Fig. 4:**
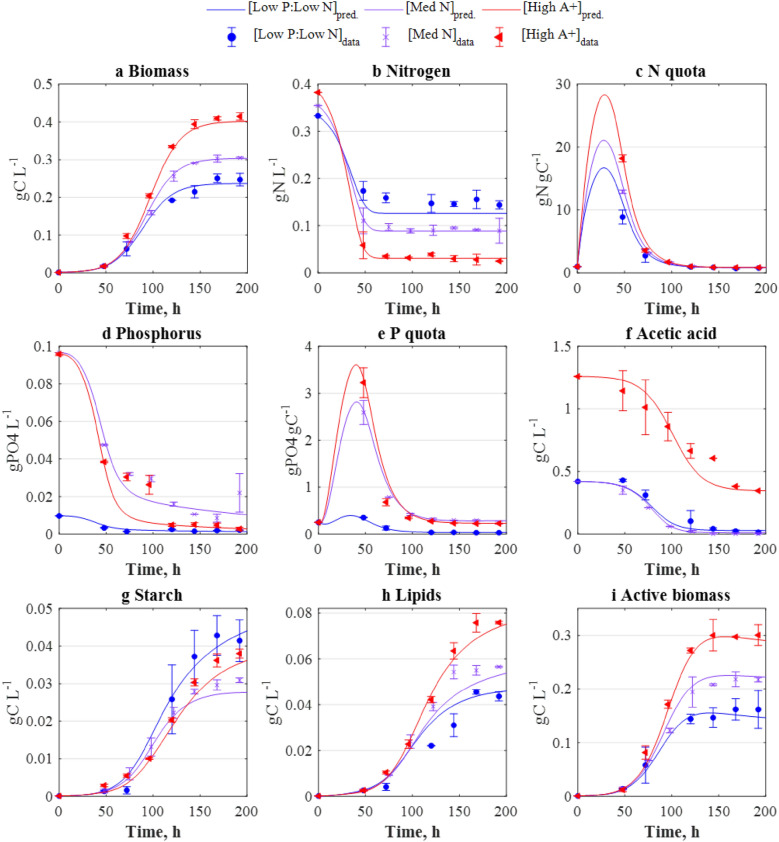
Comparison between the experimental (points) concentration–time profiles and the predictions (lines) used as model validation. (Low P: Low N): *N*_*0*_ = 0.335 gN L^−1^, *P*_*0*_ = 0.0096 gPO_4_ L^−1^, *A*_*0*_ = 0.42 gC L^−1^, [med N]: 0.354 gN L^−1^, 0.096 gPO_4_ L^−1^, 0.42 gC L^−1^, and (High A+): 0.382 gN L^−1^, 0.096 gPO_4_ L^−1^, 1.26 gC L^−1^. Data and error bars represent the mean and range (min/max values), respectively, of two independent experimental replicates

The above formulation follows the structure proposed in our previous work. However, since the consumption of nitrogen (Figs. 3b and 4b) decreased in those cultures grown in low phosphorous concentrations, the nitrogen uptake rate was additionally regulated by a Droop function of the phosphorus quota, $f({q}_{P})$:

8 $$f\left( {q_{P} } \right) = \left( {1 - \frac{{K_{P} }}{{q_{P} }}} \right).$$

Here, ${K}_{P}$ (gN gC^−1^) denotes the minimum P quota below which nitrogen uptake stops: (i.e. if ${q}_{P}<{K}_{{q}_{P}}$, ${\rho }_{N}=0$). The negative effect of phosphorus limitation on the cellular mechanisms controlling nitrogen uptake has been previously reported, and is explained by a shortage of nutrient transport energy supplied by phosphorus-containing molecules such as ATP [52].

The uptake of phosphorus, unlike nitrogen, was not affected by acetic acid and was thus solely expressed in terms of the residual phosphate concentration, *P*, by means of inhibition-type kinetics:

9 $$\rho_{P} = \rho_{{P,{\text{max}}}} \cdot \frac{P}{{P + k_{s,P} + P^{2} /k_{i,P} }} \cdot f\left( {q_{N} } \right).$$

Here, ${\rho }_{P,\mathrm{max}}$ (gPO_4_ gC^−1^ h^−1^) is the maximum phosphorous uptake rate, and ${k}_{s,P}$ and ${k}_{i,P}$ (gPO_4_ gC^−1^) are the phosphorus-associated half-saturation and inhibition constants, respectively. In Eq. (9), $f({q}_{N})$, is a regulating function dependent on the N quota which accounts for the negative effects of nitrogen stress on phosphorus uptake, described as:

10 $$f\left( {q_{N} } \right) = \left[ {1 + \left( {\frac{{\rho_{{P,{\text{max}}}} }}{{q_{N} }}} \right)^{2} } \right]^{ - 1} .$$

This function is an inverse adaptation of the classic term used for microbial product inhibition [55], but applied here to decreasing limiting N quotas. Equation (10) regulates phosphorus uptake as follows: the uptake of phosphorus decreases as the nitrogen quota decreases (i.e. nitrogen-limited conditions). The regulating function shown in Eq. (10) differs from the Droop-based function in Eq. (8) since the effect of nitrogen stress on phosphorous uptake (which reduced gradually—see Figs. 3d and 4d) was observed to be less pronounced than the effects of P-stress on nitrogen uptake (which stops abruptly). A visual comparison between the effects of the functions $f({q}_{N})$ and $f\left({q}_{P}\right)$ on the nutrient uptake rate is available in Additional file 1, Figure S2*.*

#### Formation of starch and lipids

The dynamics of starch and lipid formation are regulated by their synthetic rates, ${R}_{1}$ and ${R}_{3}$, and their degradation rates, ${R}_{2}$ and ${R}_{4}$, respectively. The synthetic rates are expressed as:

11 $$\begin{aligned} R_{1} =\, & r_{1} \cdot \frac{{N_{i}^{{n_{s} }} { }}}{{N_{i}^{{n_{s} }} + k_{s,S}^{{n_{S} }} + \left( {N_{i}^{2} /k_{i,S} } \right)^{{n_{s} }} }} \cdot \frac{{k_{1} }}{{k_{1} + N/N_{o} }} \\ &\cdot \left[ {1 + \frac{1}{\mu } \cdot e^{{\phi_{S} *A_{i} }} } \right] \cdot \mu \cdot x^{*} , \end{aligned}$$

12 $$\begin{aligned} R_{3} =\, &r_{3} \cdot \frac{{N_{i}^{{n_{L} }} { }}}{{N_{i}^{{n_{L} }} + k_{s,L}^{{n_{L} }} + \left( {N_{i}^{2} /k_{i,L} } \right)^{{n_{L} }} }} \cdot \frac{{k_{2} }}{{k_{2} + N/N_{0} }}\\ & \cdot \left[ {1 + \frac{1}{\mu } \cdot e^{{\phi_{L} *A_{i} }} } \right] \cdot \mu \cdot x^{*} . \end{aligned}$$

Here, ${r}_{1}$ (gC gC^−1^) and ${r}_{3}$ (gC gC^−1^) are the rate constants for starch and lipid synthesis, respectively. ${k}_{s,S}$ and ${k}_{s,L}$ (gN_i_ L^−1^) are saturation constants; ${k}_{i,S}$ and ${k}_{i,L}$ (gN_i_ L^−1^) are inhibition constants; ${\phi }_{S}$ and ${\phi }_{L}$ (L gC^−1^) are regulation coefficients; and ${k}_{1}$ and ${k}_{2}$ (gN L^−1^) are regulating constants. The first term in the synthetic rates is an inhibition-type function dependent on the internal nitrogen concentration, $N_{i} = q_{N} \cdot X,$ which portrays the reduced formation of storage molecules as the internal nitrogen concentration increases (i.e. nitrogen-replete conditions); the shape-controlling parameters, ${n}_{S}$ and ${n}_{L}$, are analogous to those employed in the light-limited model of Molina-Grima et al. [56], but denote here the “abruptness” of the transition from nitrogen-limited to nitrogen-replete conditions,

The second term in Eqs. (11) and (12) is a regulating function dependent on the extracellular nitrogen (scaled with respect to nitrogen supplied, i.e. *N*/*N*_*0*_) so that storage molecule formation is greater as the residual nitrogen decreases (i.e. nutrient-limited conditions). Finally, the exponential term is dependent on the bioavailable carbon concentration, i.e. ${A}_{i}={A}_{0}-A$, and simulate the increased formation of storage molecule with increasing acetic acid concentration. This increase was considered to be a result of the acetate-induced boost and therefore uncoupled from biomass growth.

The degradation rates of starch and lipids are expressed as follows:

13 $$R_{2} = r_{2} \cdot \frac{X}{{q_{N} }} \cdot \frac{S/X}{{S/X + k_{{{\text{sat}},S}} }},$$

14 $$R_{4} = r_{4} \cdot \frac{X}{{q_{N} }} \cdot \frac{L/X}{{L/X + k_{{{\text{sat}},L}} }}.$$

Here, ${r}_{2}$ and ${r}_{4}$ are the rate constants for starch and lipid degradation, respectively, and ${k}_{\mathrm{sat},S}$ and ${k}_{\mathrm{sat},L}$ (gC gC^−1^) are saturation constants. The degradation rates are assumed to be inversely proportional to the nitrogen quota to prevent excessive formation of starch and lipids during extreme nitrogen-limited conditions and maintain the pool of active biomass. The degradation rates additionally incorporate saturating functions which control the extent of starch and lipid degradation and avoid infeasible accumulation scenarios which can arise in absence of saturation (Additional file 1, Figure S3). These functions follow the formulation proposed by Contois [57].

Whilst the synthetic and degradation rates presented above were developed to portray the individual dynamics of starch and lipid during nutrient-limited mixotrophic growth observed in this work, other kinetic expressions have been reported to describe the substrate-to-product interactions leading to starch and lipid formation under other cultivation conditions, including autotrophic growth, limitation by a single nutrient, or light and temperature limitations [34].

#### Time-dependent equations

The accumulation rates of the carbon-based cell components (i.e. biomass, starch, lipids, and active biomass) are described by the following set of ordinary differential equations:

15 $$\frac{{{\text{d}}X}}{{{\text{d}}t}} = \mu \cdot X,$$

16 $$\frac{{{\text{d}}S}}{{{\text{d}}t}} = R_{1} - R_{2} ,$$

17 $$\frac{{{\text{d}}L}}{{{\text{d}}t}} = R_{3} - R_{4} ,$$

18 $$\frac{{{\text{d}}x^{*} }}{{{\text{d}}t}} = \frac{{{\text{d}}X}}{{{\text{d}}t}} - \left( {\frac{{{\text{d}}S}}{{{\text{d}}t}} + \frac{{{\text{d}}L}}{{{\text{d}}t}}} \right).$$

The extracellular and intracellular (i.e. cell quotas) nutrient dynamics are described by:

19 $$\frac{{{\text{d}}N}}{{{\text{d}}t}} = - \rho_{N} \cdot X,$$

20 $$\frac{{{\text{d}}q_{N} }}{{{\text{d}}t}} = \rho_{N} - \mu \cdot q_{N} ,$$

21 $$\frac{{{\text{d}}P}}{{{\text{d}}t}} = - \rho_{P} \cdot X,$$

22 $$\frac{{{\text{d}}q_{P} }}{{{\text{d}}t}} = \rho_{P} - \mu \cdot q_{P} ,$$

23 $$\frac{{{\text{d}}A}}{{{\text{d}}t}} = - \frac{1}{{Y_{X/A} }} \cdot \frac{{\mu_{H} }}{{\mu_{H} + \mu_{I} }} \cdot \frac{{{\text{d}}X}}{{{\text{d}}t}}.$$

In Eq. (23), Y_X/A_ (gC gC^−1^) is the acetate to biomass yield coefficient.

### Evaluating the model’s predictive performance

The multi-parametric model proposed above [Eq. (15)–Eq. (23)] comprised 37 kinetic parameters (Table 2), which were estimated through a data fitting procedure combining deterministic and stochastic algorithms. The fitting procedure was then followed by a normalised sensitivity analysis to evaluate the response change in a model state variable with respect to a 1% change in the parameter values (Additional file 1. Figure S6). As a result of this analysis, model parameters were reduced to 35. The model was then evaluated in terms of its capacity to predict microalgal growth dynamics subject to different nitrogen, phosphorus, and acetic acid concentrations.

**Table 2:** List of parameters in the model for mixotrophic growth co-limited by nitrogen and phosphorus

Type	Symbol	Parameter description	Value	Units	References
Associated to growth	*µ*_max_	Maximum specific growth rate	0.106	h^−1^	Figueroa-Torres et al. [42]
	*q*_*N,0*_	Minimum nitrogen quota	0.877	gN gC^−1^	Figueroa-Torres et al. [42]
	*q*_*P,0*_	Minimum phosphorus quota	0.016	gPO_4_ gC^−1^	This work
	*K*_*s,A*_	Acetate saturation constant	1.789	gC L^−1^	Figueroa-Torres et al. [42]
	*k*_*i,A*_	Acetate inhibition constant	0.110	gC L^−1^	Figueroa-Torres et al. [42]
	*K*_*s,I*_	Light saturation constant	1.4	µ_mol_ m^−2^ s^−1^	Mairet et al. [53]
	*k*_*i,I*_	Light inhibition constant	186.5	µ_mol_ m^−2^ s^−1^	Figueroa-Torres et al. [42]
	*Y*_*X/A*_	Acetate yield coefficient	0.059	gC gC^−1^	Figueroa-Torres et al. [42]
	*Ϭ*	Light attenuation coefficient	1	L gC^−1^ m^−1^	Figueroa-Torres et al. [42]
Associated to N and P -uptake	*ρ*_*N,*max_	Maximum N uptake rate	44.01	gN gC^−1^ h^−1^	This work*
	K_*_	Saturation constant, *N*_*o*_	0.300	gN L^−1^	This work*
	*n*	Shape-controlling parameter	14.54	–	This work*
	Ф_*N*_	N Uptake regulation coefficient	143.9	L gC^−1^	This work*
	*K*_*s,N*_	Uptake saturation constant, *N*	0.163	gN L^−1^	Figueroa-Torres et al. [42]
	*k*_*i,N*_	Uptake inhibition constant, *N*	0.113	gN L^−1^	Figueroa-Torres et al. [42]
	*K*_*s,A:N*_	Uptake saturation constant, *A:N*	1.004	gC L^−1^	Figueroa-Torres et al. [42]
	*k*_*i,A:N*_	Uptake inhibition constant, *A:N*	1.098	gC L^−1^	Figueroa-Torres et al. [42]
	*K*_*P*_	P quota supporting *N* uptake	0.057	gPO_4_ gC^−1^	This work
	*ρ*_*P,*max_	Maximum P uptake rate	21.10	gPO_4_ gC^−1^ h^−1^	This work
	*K*_*s,P*_	Uptake saturation constant, *P*	2.299	gPO_4_ L^−1^	This work
	*k*_*i,P*_	Uptake inhibition constant, *P*	0.004	gPO_4_ L^−1^	This work
Associated to starch and lipid formation	*r*_*1*_	Starch formation rate (*R*_*1*_)	0.058	gC gC^−1^	This work*
	*K*_*s,S*_	Saturation constant (*R*_*1*_)	0.000	gN L^−1^	This work*
	*k*_*i,S*_	Inhibition constant (*R*_*1*_)	0.205	gN L^−1^	This work*
	*n*_*S*_	Shape parameter (*R*_*1*_)	4.17	–	This work*
	*k*_*1*_	Regulation constant (*R*_*1*_)	0.108	–	This work*
	*Ф*_*S*_	Regulation coefficient (*R*_*1*_)	0.775	L gC^−1^	This work*
	*r*_*2*_	Starch degradation rate (*R*_*2*_)	0.005	gC gC^−1^	This work*
	*k*_sat*,S*_	Starch saturation constant (*R*_*2*_)	0.018	–	This work
	*r*_*3*_	Lipid formation rate (*R*_*3*_)	0.191	gN gC^−1^ h^−1^	This work*
	*K*_*s,L*_	Saturation constant (*R*_*3*_)	0.012	gN L^−1^	This work*
	*k*_*i,L*_	Inhibition constant (*R*_*3*_)	0.091	gN L^−1^	This work*
	*n*_*L*_	Shape parameter (*R*_*3*_)	2.01	–	This work*
	*k*_*2*_	Regulation constant (*R*_*3*_)	0.153	–	This work*
	*Ф*_*L*_	Regulation coefficient (*R*_*3*_)	0.000	L gC^−1^	This work*
	*r*_*4*_	Lipid degradation rate (*R*_*4*_)	0.007	gN gC^−1^ h^−1^	This work*
	*k*_sat*,L*_	Lipid saturation constant (*R*_*4*_)	0.079	–	This work

^*^ Parameter values were re-identified from those established in Figueroa-Torres et al. [42]

As shown in Fig. 3, the concentration–time profiles obtained by the model were observed to be in good agreement with the corresponding experimental datasets used for parameter estimation. The model outcomes were subsequently validated against different cultivation regimes, similarly yielding a good agreement between model-predicted and experimental data and (Fig. 4). Parity plots showing the level of agreement between experimental and model-derived data can be found in the Additional file 1: Figure S4. The computed mean correlation coefficient (*r*^*2*^) between the experimental and model-derived (fitting and validation) datasets averaged *r*^*2*^ = 0.95, highlighting the model’s high predictive capacity, and indicating that the model adequately portrays growth, nutrient uptake, and starch and lipid formation in *C. reinhardtii*.

It is worth mentioning here that despite the robustness of the model for a wide range of environmental conditions, established through extensive experimental validation, the relatively large number of kinetic parameters incorporated might lead to identifiability issues, requiring additional work to compute the “true” optimal values of the model parameters.

**Fig. 5:**
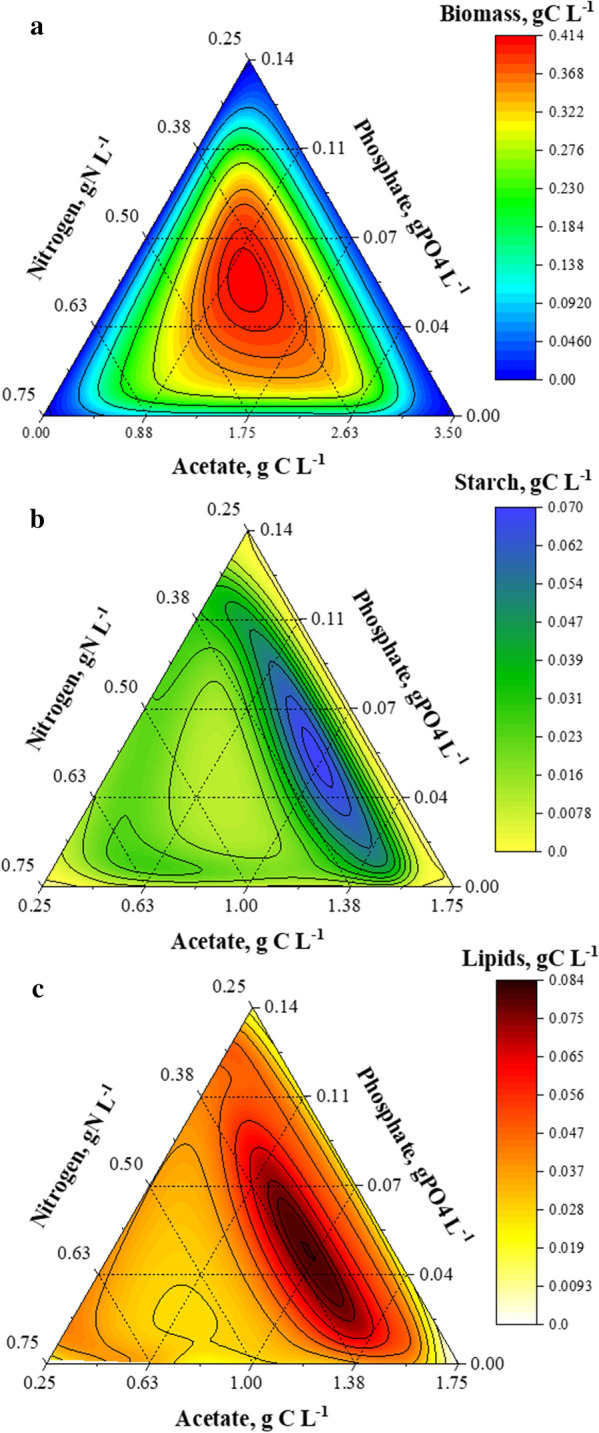
Ternary diagrams for: **a** biomass, **b** starch, and **c** lipid formation in *C. reinhardtii.* Diagrams reflect metabolites concentration at *t* = 192 h, as predicted by the model, when subject to different initial nitrogen, phosphorus, and acetic acid concentration sets

The model was then exploited to compute the formation of biomass, starch, and lipids at the 8th day (*t* = 192 h) of cultivation, subject to various initial nitrogen (0.25–0.75 gN L^−1^), phosphorus (0–0.14 gPO_4_ L^−1^), and acetic acid (0–3.5 gC L^−1^) concentrations. The results are presented as three individual ternary diagrams (Fig. 5), each showing predicted biomass, starch, and lipids (model outputs) in response to initial nutrient concentrations (model inputs). The ternary diagrams show the corresponding changes in starch and lipid formation when subject to nitrogen and phosphorus co-limitation, and allow identification of the required nutrient characteristics to maximise starch and lipid formation during acetate-driven mixotrophic growth.

### Maximising microalgal starch and lipid formation

**Fig. 6:**
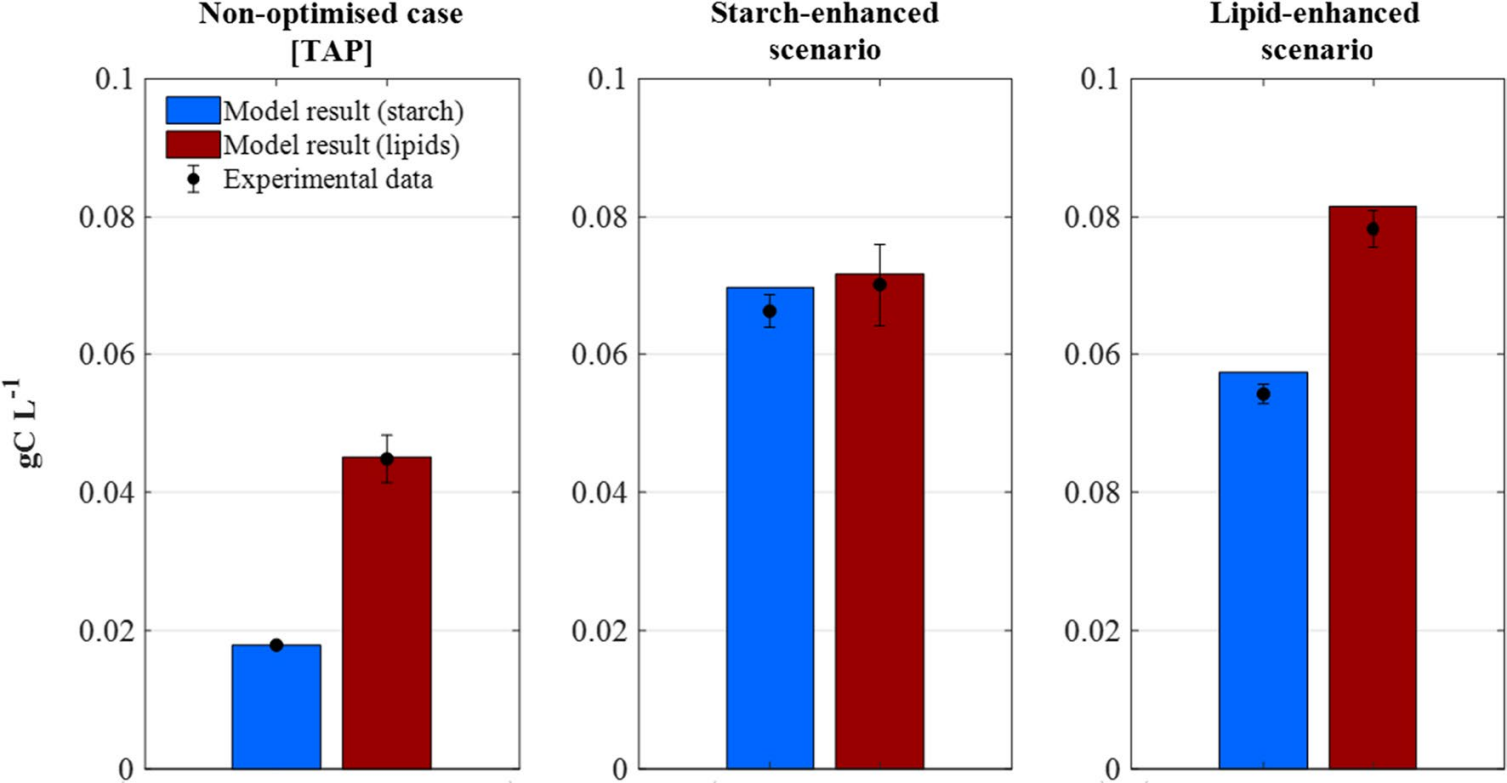
Comparison of model results and experimental data (dots) from the non-optimised, and model-based optimised scenarios for maximised starch and lipid formation. Non-optimised medium (*N*_*o*_ = 0.382 gN L^−1^, *P*_*0*_ = 0.096 gPO_4_ L^−1^, *A*_*0*_ = 0.42 gC L^−1^), starch-enhancing medium (*N*_*0*_ = 0.33 gN L^−1^, *P*_*0*_ = 0.052 gPO_4_ L^−1^, *A*_*0*_ = 0.96 gC L^−1^), and lipid-enhancing medium (*N*_*0*_ = 0.35 gN L^−1^, *P*_0_ = 0.044 gPO_4_ L^−1^, *A*_*0*_ = 0.96 gC L^−1^). Data and error bars represent the mean and range (min/max values), respectively, of two independent experimental replicates

The ternary diagrams were employed to identify the optimal nutritional requirements (i.e. nitrogen, phosphorus, and acetic acid) maximising starch and lipid concentrations, identified as: (i) “*starch-enhancing*” medium: [*N*_*o*_ = 0.33 gN L^−1^, *P*_*0*_ = 0.052 gPO_4_ L^−1^, *A*_*o*_ = 0.96 gC_A_ L^−1^], yielding 0.33 gC L^−1^ biomass with 21% starch and 22% lipids, and (ii) “*lipid-enhancing*” medium [*N*_*o*_ = 0.35 gN L^−1^, *P*_*0*_ = 0.044 gPO_4_ L^−1^, *A*_*o*_ = 0.96 gC L^−1^], yielding 0.38 gC L^−1^ biomass with 15% starch and 21% lipids. The predicted outcome of the optimised scenarios was additionally verified by growing two lab-scale cultures of *C. reinhardtii* subject to the above optimal medium compositions. As observed in Fig. 6, both of the model-based optimal cultivation scenarios agreed well with the corresponding experimental data. Compared to (TAP) medium, *starch-enhancing* conditions yielded increases of 270% and 56% in starch and lipid concentrations, respectively, whereas *lipid-enhancing* conditions yielded increases of 203% and 74% in starch and lipid concentrations, respectively.

## Discussion

Many species of microalgae respond to nutrient limitation by significantly altering central carbon metabolism pathways and intracellular carbon partitioning, leading to compositional changes which generally favour accumulation of storage molecules [24, 33, 58]. As observed in Table 1, nitrogen and phosphorus limitation resulted in greater starch and lipids contents. Although nitrogen and phosphorus limitation are among the most extensively proven cultivation strategies for starch and lipid accumulation, studies have mainly evaluated such strategies under either complete starvation or single-nutrient limitation [24, 27, 59–61]. Few works evaluate the accumulation of storage molecules under different degrees of nutrient co-limitation [26, 28] which is characterised by a trade-off between microalgal growth and starch and lipid accumulation. As evidenced here, however, such a trade-off was overcome by the gradual increase of acetic acid (i.e. the mixotrophic carbon source) which resulted in higher biomass production and, consequently, higher starch and lipid production.

Nutrient-limited mixotrophic cultivation is thus a suitable cultivation strategy for the purposes of biofuels production, but its implementation is dependent on the identification of an optimal nutrient composition. The multi-parametric kinetic model presented here, developed through a combination of experimental and computational tools, was shown to be a robust tool for the simulation of mixotrophic microalgal growth subject to a wide range of nutrient compositions (Figs. 3 and 4). The developed model was thus further exploited to identify *starch-enhancing* and *lipid-enhancing* cultivation strategies and, when compared to a non-optimised scenario, the model-identified strategies yielded significant increases of + 270% starch and + 74% lipids.

In line with these optimal scenarios, co-limitation by nitrogen and phosphorus can significantly induce starch and lipid formation, but provided that reduced growth rates are overcome via the supply of sufficient acetic acid, i.e. carbon source. Both nitrogen and phosphorous are essential nutrients that make up important biomolecules such as proteins, pigments, phospholipids, nucleic acids, RNA and DNA, which regulate vital metabolic processes (e.g. the photosynthetic pathway by which carbon is fixated) [24, 62]. Therefore, the enhanced effect of acetic acid supplementation would only be attained if the concentrations of nitrogen and phosphorous allow for growth to take place. As observed in the ternary diagrams shown in Fig. 5, such optimal conditions can be established at the onset of batch cultivation. However, it is worth noting that other operating strategies involving fed-batch or continuous operation will require a balanced supply of nutrients preventing their exhaustion in the cultivation medium.

From an economic perspective the organic carbon requirements may restrict mixotrophic cultivation, but this could be avoided by adequately integrating wastewater effluents rich in organic matter with microalgal growth [63, 64]. The validated optimal nutrient compositions identified here thus offer a promising and sustainable outlook for the scaling-up of microalgal cultivation systems for biorefinery applications where, on one hand, biofuel precursor molecules are maximised and, on the other, nutrient supply is efficiently and sustainably managed (for instance, by reducing the environmental impacts of nitrogen fertilisers or the overuse of inorganic phosphorus, a non-renewable resource [65]).

## Conclusions

The multi-parametric kinetic model presented here, developed through a combination of experimental and computational tools, was shown to be a robust tool for the simulation of mixotrophic microalgal growth subject to a wide range of nutrient compositions. The developed model was further exploited to identify *starch-enhancing* and *lipid-enhancing* cultivation strategies relying on optimal nutrient composition. When compared to a non-optimised scenario, the model-identified strategies yielded significant increases of + 270% starch and + 74% lipids. Establishing highly productive microalgal cultivation strategies is one of the major challenges preventing microalgal biomass to be implemented as feedstock for biofuels production. However, the model-based optimisation framework presented in this work can be systematically applied to identify and implement tailor-made cultivation strategies yielding mass-scale microalgal cultures rich in starch and lipids and thus contribute to the commercialisation of microalgal biofuels, and together with on-going technological advances, the establishment of microalgal biorefineries.

## Methods

### Strain and cultivation

Experiments were carried out with the wild-type strain *Chlamydomonas reinhardtii* CCAP 11/32C. The strain was grown mixotrophically in Tris–Acetate–Phosphate (TAP) medium [35]: 2.42 g of Tris-base, 25 mL of TAP salts (15 g L^−1^ NH_4_Cl, 4 g L^−1^ MgSO_4_⋅7H_2_O, 2 g L^−1^ CaCl_2_⋅2H_2_O), 0.387 mL of phosphate buffer 2.7 M (288 g L^−1^ K_2_HPO_4_, 144 g L^−1^ KH2PO_4_), 1 mL of trace components [66], and 1 mL of acetic acid, brought to 1 L with deionised water. For nutrient-dependent experiments a microalgal inoculum was propagated in 150 mL of TAP medium until the late stationary phase (5–7 days), reaching a cell dry weight of 0.001 g mL^−1^ (5.47 × 10^6^ cells mL^−1^). The inoculum was placed in an orbital shaker at 150 rpm, 25 °C. Illumination (at 125 μmol m^−2^ s^−1^) was provided from above using 4 ft long 20 W high-power LED T8 tube lights in a light/dark photoperiod of 16/8 h, and the length of light path was 0.15 m.

### Nutrient-dependent cultures

Mixotrophic growth dynamics co-limited by nitrogen and phosphorus were evaluated by growing microalgal cultures under different initial nitrogen (*N*_*0*_), phosphorus (*P*_*0*_), and acetic acid (*A*_*0*_) concentrations with respect to standard (TAP) medium (Table 1). Cultures were grown in duplicate in 500 mL of sterile medium, inoculated with 1 mL of active microalgal inoculum, and kept at the environmental conditions described above. Cultures were fully harvested during cultivation (days 2, 3, 4, 6, 7, and 8) to analyse biomass and metabolites. Data was statistically analysed by one-way ANOVA in Origin Pro 2017 (b9.4.1.354).

During media preparation, the initial nitrogen concentration was altered by modifying the concentration of ammonium chloride (NH_4_Cl) in the TAP salts solution. Initial phosphorus concentration was altered by modifying accordingly the volume of phosphate buffer (maintaining a 2:1 ratio for K_2_HPO_4_:KH_2_PO_4_). In phosphorus-limited media, potassium chloride (KCl) was uniformly added to compensate for the loss of potassium ions. Initial acetic acid concentration was altered by modifying the volume of acetic acid. The concentration of all other TAP components remained unchanged, and the initial medium pH was adjusted to 7 with HCl 3 M or KOH 3 M, as appropriate.

## Analytical methods

### Cell growth

The dry cell weight (DCW) was quantified by centrifuging microalgal cultures for 3.5 min at 3000*g* in an Eppendorf centrifuge 5424. The residual cell pellets were placed in pre-weighed tubes and allowed to dry for 24 h at 70 °C, after which the DCW was determined gravimetrically. Dried pellets were kept in sealed containers and analysed for their lipid content.

### Starch and lipid contents

For analysis of microalgal starch, 2-mL aliquot samples of microalgal cultures were pelleted by centrifugation at 13,000*g* for 3 min. Chlorophyll was removed by washing pelleted cells in 500 μL of 80% ethanol for 5 min at 85 °C. Washed cells were re-centrifuged at 13,000*g* for 3 min, and cellular starch was then solubilised as described in Bajhaiya et al. [26]). Total starch was quantified as per a Total Starch enzymatic assay kit (Megazyme International) where released free D-glucose is measured colourimetrically against a d-glucose standard curve. The lipid content of cells (previously pulverised) was determined by solvent extraction (using hexane at 155 °C) in a SOXTEC Unit 1043 following a three-stage extraction protocol [31]. Extracted lipids were then quantified gravimetrically.

### Metabolites concentrations

Acetic acid was quantified by high-pressure liquid chromatography (HPLC) in a HPX-87H column (8 μm, 300 × 7.7 mm, Bio-Rad), coupled to a UV detector set at 210 nm. Sulphuric acid (H_2_SO_4_) 5 μM was used as the mobile phase at a flow rate of 0.6 mL min^−1^ and a temperature of 50 °C. Total nitrogen was measured in a Total Organic Carbon/Total Nitrogen unit (TOC-V_CSH_/TNM-1 Shimadzu) as per manufacturer’s instructions. For calibration standards, ammonium chloride (NH_4_Cl) was used as the nitrogen source. Phosphorus was measured by Inductively Coupled Plasma—Optical Emission Spectroscopy (ICP-OES) in a Varian Vista MPX set at 213 nm. All samples and calibration standards were filtered through 0.45 μm nitrocellulose membranes (Millipore Ltd.) and diluted accordingly in Type 1 grade water. The nitrogen and phosphorus cellular quotas were estimated as follows:

24 $$q_{N} = \frac{{N_{o} - N}}{X};q_{P} = \frac{{P_{o} - P}}{X},$$

where *N*_*0*_ (gN L^−1^) and *P*_*0*_ (gPO_4_ L^−1^) are the initial nitrogen and phosphorus medium concentrations, respectively, and *N*, *P*, and *X* are the residual concentrations of nitrogen, phosphorus, and biomass, respectively [52].

### Active biomass and carbon equivalent concentration

The fraction of active biomass (i.e. starch and lipid free biomass) was determined by subtracting starch and lipid concentration from the total biomass (DCW). Acetic acid, starch, lipids, and biomass are reported on a carbon basis by means of conversion factors (gC g^−1^): 0.40 acetate, 0.44 starch, 0.77 lipids, and 0.504 biomass. *C. reinhardtii* cells were assumed to have the elemental composition reported by Eriksen et al. [67].

### Estimation of model parameters

The model presented in this work [Eqs. (16)–(24)] is comprised by 37 kinetic parameters, all appropriately defined in Table 2. The values of 12 kinetic parameters (associated to growth and nitrogen uptake dynamics) were set equivalent to those previously identified by Figueroa-Torres et al. [42]. The remaining kinetic parameters were estimated by minimising the squared relative error between experimental and predicted data:

25 $$\min G\left( P \right) = \mathop \sum \limits_{h = 1}^{nh} \mathop \sum \limits_{i = 1}^{ni} \mathop \sum \limits_{k = 1}^{nk} \left( {\frac{{Z_{h,i,k}^{{{\text{Pred}}}} \left( P \right) - Z_{h,i,k}^{{{\text{Exp}}}} }}{{Z_{h,i,k}^{{{\text{Exp}}}} }}} \right)^{2} .$$

Here, $G$ is the objective function, *P* is the parameter set, and *Z* is the set of predicted or experimental data. Predicted data were generated by solving the model using initial values equivalent to those of nutrient-dependent experiments. *nh*, *ni*, and *nk* denote the number of data points in time, number of fitting experimental datasets [3 datasets: (TAP), (Low P), and (High A: Low P)], and number of state variables, respectively. Parameters were restricted by lower (*lb*) and upper (*ub*) bounds as per data obtained from literature or experimental analysis. The minimisation problem was solved via a stochastic optimisation routine (simulated annealing) subject to multiple re-starts to approximate the solution around a global minimum. The stochastic solution was then used as initial guess in a deterministic routine (sequential quadratic programming) to generate the final parameter set [68]. Both routines were coded in-house in Matlab R2015a. A sensitivity analysis was carried out for all model parameters and is presented as Additional file 1. As per the sensitivity analysis, 4 parameters were deemed not sensitive ($\sigma$, ${k}_{S,I}$, ${K}_{s,S}$, and ${\phi }_{L}$): from which two parameters were set as $\sigma =1$, and ${k}_{S,I}=1.4$, and the other two were found to have a negligible effect on model predictions when set to 0, so that ${K}_{s,S}=0$, and ${\phi }_{L}=0$.

## Supplementary Information

**Additional file 1.** **Table S1**. Initial nutrient concentrations employed during nutrient-dependent cultures. **Table S2**. p-values obtained by one-way ANOVA (tukey test). Asterisks (*) denote significant differences (p <0.05*, 0.01**, 0.001***) with respect to (TAP). The analysis was carried out in Origin Pro 2017 (b9.4.1.354). **Table S3**. Experimental data obtained in *C. reinhardtii* cultures grown in varying initial acetic acid concentration, with constant No=0.382 gN L-1. **Table S4**. Experimental data obtained in *C. reinhardtii* cultures grown in varying initial nitrogen concentration, with constant Po=0.96 gPO4 L-1. **Figure S1**. Comparison between the model-derived (lines) and experimental (points) data for various *C. reinhardtii* grown under different initial acetic acid concentrations (No=0.3824 gN L-1, Po=0.096 gPO4 L-1). Data and simulation corresponds to t=192 h. **Figure S2**. Visual comparison between the effects of the two quota-dependent functions on the nutrient uptake rates used in the model. **Figure S3**. Cultivation dynamics subject to three cultivation conditions, simulated by the a) “saturated” model, and the b) “unsaturated” model. **Figure S4**. Parity plots comparing predicted and experimental data for both fitting and validating datasets for: a) biomass, b) nitrogen, c) nitrogen quota, d) phosphorus, e) phosphorus quota, f) acetic acid, g) starch, h) lipids, and i) active biomass. Data are the mean of two independent experimental replicates. **Figure S5**. Normalised sensitivity of the model state variables with respect to a 1 % increase in , over a 200 h cultivation period subject to different initial phosphorous concentrations. Parameter colour denotes: green – associated to biomass growth. **Figure S6**. Normalised sensitivity of the model state variables with respect to a 1 % increase in each model parameter, over a 200 h cultivation period. Parameter colours denote: green – associated to biomass growth, purple – associated to N uptake, orange – associated to P uptake, and black – associated to starch and lipid formation.

## Data Availability

The main data supporting the findings of this study are available within the article and its additional information. Additional data are available from the corresponding authors on reasonable request.
