# Supplementary material for: Optimisation of microalgal cultivation via nutrient-enhanced strategies: the biorefinery paradigm
Source: Biotechnol Biofuels. 2021 Mar 12;14:64. doi: 10.1186/s13068-021-01912-2 (PMC7953610; doi:10.1186/s13068-021-01912-2)
Supplement: Supplementary file 1 — Additional file 1. Table S1. Initial nutrient concentrations employed during nutrient-dependent cultures. Table S2. p-values obtained by one-way ANOVA (tukey test). Asterisks (*) denote significant differences (p <0.05*, 0.01**, 0.001***) with respect to (TAP). The analysis was carried out in Origin Pro 2017 (b9.4.1.354). Table S3. Experimental data obtained in C. reinhardtii cultures grown in varying initial acetic acid concentration, with constant No=0.382 gN L-1. Table S4. Experimental data obtained in C. reinhardtii cultures grown in varying initial nitrogen concentration, with constant Po=0.96 gPO4 L-1. Figure S1. Comparison between the model-derived (lines) and experimental (points) data for various C. reinhardtii grown under different initial acetic acid concentrations (No=0.3824 gN L-1, Po=0.096 gPO4 L-1). Data and simulation corresponds to t=192 h. Figure S2. Visual comparison between the effects of the two quota-dependent functions on the nutrient uptake rates used in the model. Figure S3. Cultivation dynamics subject to three cultivation conditions, simulated by the a) “saturated” model, and the b) “unsaturated” model. Figure S4. Parity plots comparing predicted and experimental data for both fitting and validating datasets for: a) biomass, b) nitrogen, c) nitrogen quota, d) phosphorus, e) phosphorus quota, f) acetic acid, g) starch, h) lipids, and i) active biomass. Data are the mean of two independent experimental replicates. Figure S5. Normalised sensitivity of the model state variables with respect to a 1 % increase in , over a 200 h cultivation period subject to different initial phosphorous concentrations. Parameter colour denotes: green – associated to biomass growth. Figure S6. Normalised sensitivity of the model state variables with respect to a 1 % increase in each model parameter, over a 200 h cultivation period. Parameter colours denote: green – associated to biomass growth, purple – associated to N uptake, orange – associated to P uptake, and [file 13068_2021_1912_MOESM1_ESM.docx]

**Additional file 1**

1. **Nutrient-dependent experiments.**

Table S1. Initial nutrient concentrations employed during nutrient-dependent cultures.

| **Treatment Label** | **Nitrogen ^a^** | **Phosphorus** | **Acetic acid** | |
| --- | --- | --- | --- | --- |
|  | **gN L^-1^  (gN L^-1^)** | **gPO_4_ L^-1^** | **gC L^-1^** | |
| **TAP *^b^*** | **0.382 (0.098)** | **0.096** | **0.42** | |
| **[Low P : Low N]** | 0.335 (0.042) | 0.0096 | 0.42 | |
| **[Low N]** | 0.335 (0.042) | 0.0960 | 0.42 | |
| **[Med N]** | 0.356 (0.070) | 0.0960 | 0.42 | |
| **[Low P]** | 0.382 (0.098) | 0.0096 | 0.42 | |
| **[Med P]** | 0.382 (0.098) | 0.0480 | 0.42 | |
| **[Low A]** | 0.382 (0.098) | 0.0960 | 0.21 | |
| **[High A]** | 0.382 (0.098) | 0.0960 | 0.75 | |
| **[High A +]** | 0.382 (0.098) | 0.0960 | 1.26 | |
| **[High A : Low N-]** | 0.315 (0.032) | 0.0960 | 1.26 | |
| **[High A : Low P]** | 0.382 (0.098) | 0.0096 | 1.26 | |
| **[High N++]** | 0.742 (0.450) | 0.0960 | 0.42 | |
| **[High P++]** | 0.382 (0.098) | 0.3860 | 0.42 | |
| **[High A++]** | 0.382 (0.098) | 0.0960 | 2.52 | |
| *^a^ First column refers to total nitrogen concentration; second column refers only to the nitrogen concentration from NH_4_Cl.* | | | |  |
| *^b^ Initial nutrient concentrations in standard TAP medium.* | | | |  |

**Table S2. p-values obtained by one-way ANOVA (tukey test). Asterisks (*) denote significant differences (p <0.05*, 0.01**, 0.001***) with respect to [TAP].** **The analysis was carried out in Origin Pro 2017 (b9.4.1.354).**

|  | **p values** | | | | |
| --- | --- | --- | --- | --- | --- |
| **Treatment pair** | **X** | **S (%)** | **S (gC/L)** | **L (%)** | **L (gC/L)** |
| [TAP] | - | - | - | - | - |
| [TAP] [Low P : Low N] | 0.0483* | 4.24E-06*** | 0.00E+00*** | 0.1524 | 1.0000 |
| [TAP] [Low N] | 0.7886 | 4.73E-06*** | 0.00E+00*** | 0.0007 | 0.0179* |
| [TAP] [Med N] | 1.0000 | 0.0398* | 8.47E-06*** | 0.0429* | 0.0883 |
| [TAP] [Low P] | 0.3837 | 0.0063** | 1.70E-05*** | 0.9720 | 0.9970 |
| [TAP] [Med P] | 0.9850 | 0.9764 | 0.6502 | 1.0000 | 0.9992 |
| [TAP] [Low A] | 0.2189 | 1.0000 | 0.0684 | 1.0000 | 0.7367 |
| [TAP] [High A] | 0.0438* | 1.0000 | 0.2327 | 0.2928 | 0.0005 |
| [TAP] [High A+] | 0.0099** | 0.1675 | 2.97E-08*** | 0.0628 | 8.99E-06 |
| [TAP] [High A : Low N-] | 0.0269* | 0.00E+00*** | 0.00E+00*** | 0.0021** | 0.9989 |
| [TAP] [High A : Low P] | 0.3421 | 0.5626 | 1.37E-05*** | 0.5414 | 0.0052** |
| [TAP] [HIGH N] | 0.0001*** | 0.4311 | 0.3261 | 1.0000 | 0.0009 |
| [TAP] [HIGH P] | 0.9842 | 1.0000 | 0.8638 | 1.0000 | 1.0000 |
| [TAP] [HIGH A] | 0.9838 | 1.0000 | 1.0000 | 1.0000 | 0.9996 |

1. **Model building**

***Inhibition of biomass growth and N consumption by acetic acid.***

As observed in Table S3, experimental data suggested that a high acetic acid concentration became inhibitory for biomass growth and reduced nitrogen consumption. Therefore, acetate-dependent inhibition functions were incorporated into the specific growth (eq. 1 in the text) and nitrogen uptake (eq. 6 in the text) rates of the model.

**Table S3. Experimental data obtained in *C. reinhardttii* cultures grown in varying initial acetic acid concentration, with constant No=0.382 gN L^-1^.**

|  |  | **Data at t=192 h** | | | |
| --- | --- | --- | --- | --- | --- |
|  | **Ao gC L^-1^** | **Biomass gC L^-1^** | **Residual N gN L^-1^** | **N consumed gN L^-1^** | **Δ N consumed** |
| **[TAP]** | 0.42 | 0.318 | 0.088 | 0.294 | - |
| **[High A]** | 0.75 | 0.390 | 0.045 | 0.337 | 15% |
| **[High A+]** | 1.26 | 0.414 | 0.025 | 0.357 | 21% |
| **[High A++]** | 2.52 | 0.294 | 0.080 | 0.302 | 3% |

**
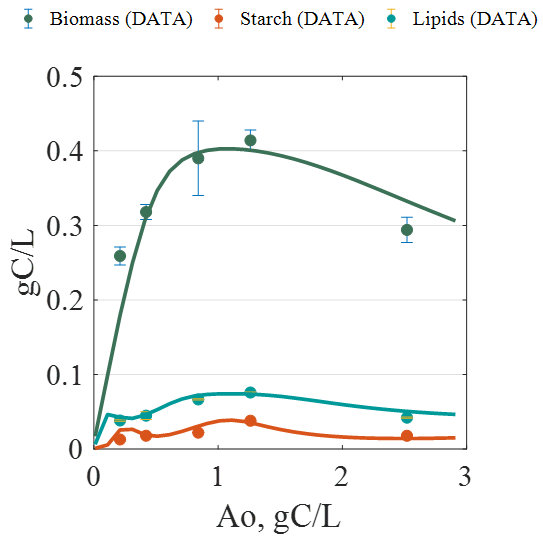
**

**Figure S1.** Comparison between the model-derived (lines) and experimental (points) data for various *C. reinhardtii* grown under different initial acetic acid concentrations (*N_o_*=0.3824 gN L^-1^, *P_o_*=0.096 gPO_4_ L^-1^). Data and simulation corresponds to *t*=192 h.

***Effect of nitrogen limitation on phosphorous consumption***

Experimental data (Table S4 below) similarly indicated that nitrogen limitation reduced phosphorous consumption.

**Table S4. Experimental data obtained in *C. reinhardttii* cultures grown in varying initial nitrogen concentration, with constant Po=0.96 gPO_4_ L^-1^.**

|  |  | **Data at t=192 h** | | | |
| --- | --- | --- | --- | --- | --- |
|  | **No gN L^-1^** | ***q_N_* gN gC^-1^** | **Residual P gPO_4_ L^-1^** | **P consumed gPO_4_ L^-1^** | **Δ P consumed** |
| **[TAP]** | 0.382 | 0.92 | 0.013 | 0.083 | - |
| **[Med N]** | 0.356 | 0.87 | 0.022 | 0.074 | -11% |
| **[Low N]** | 0.335 | 0.80 | 0.025 | 0.071 | -14% |

The effect of P-limitation on nitrogen uptake was simulated by a simple Droop function of the phosphorous quota:

$f\left( q_{P} \right)=\left( 1-\frac{K_{P}}{q_{P}} \right)$ (Eq. 8 in the text)

This Droop function, however, failed to replicate the negative effect of N-limitation on phosphorous uptake. Therefore, the following inhibition type-function was instead used in the model:

$f(q_{N})=\left[ 1+\left( \frac{\rho_{P,max}}{q_{N}} \right)^{2} \right]^{-1}$ (Eq. 10 in the text)

This inhibitory function is a derivation of the classic term used to describe microbial product inhibition (Levenspiel, 1980):

$$f\left( C_{P} \right)=\left( 1-\frac{C_{P}}{C_{P}^{*}} \right)^{n}$$

where C_P_ is the product concentration and C_P_* is the limiting product concentration above which cellular growth stops. Eq. 10 is an inverse adaptation of this classic inhibition function but applied to a decreasing N quota, rather than to an increasing product concentration.

Figure S1 below shows a visual comparison of the quota-dependent functions employed in this model: $f(q_{N})$ (eq. 10) simulates a gradual reduction in the nutrient uptake rate until the quota reduces to a zero value; the Droop function $f\left( q_{P} \right)$ (eq. 8), on the contrary, simulates the gradual reduction in nutrient uptake rate but stopping at a finite nutrient quota.


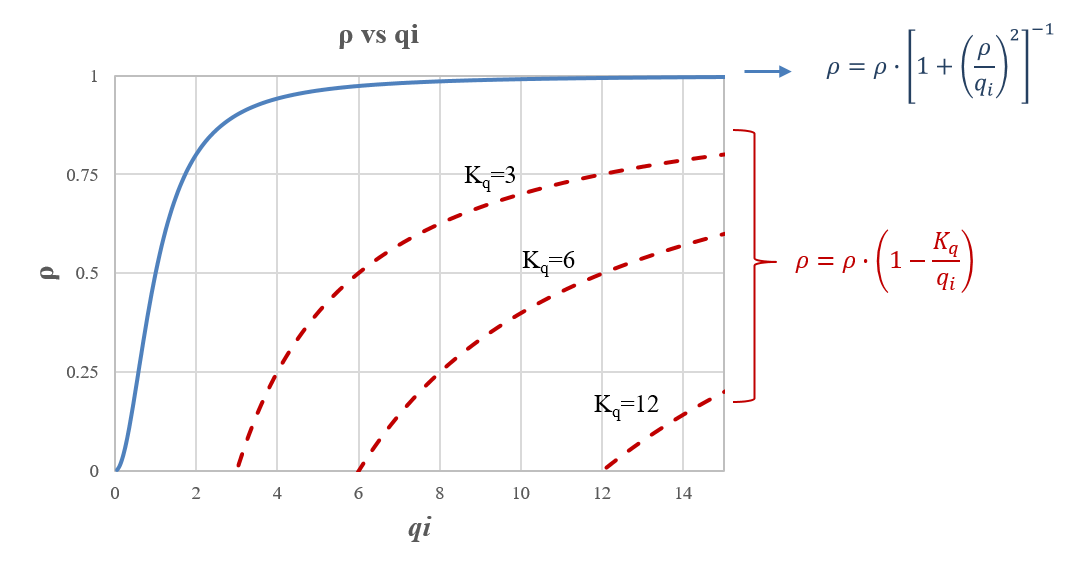


**Figure S2. Visual comparison between the effects of the two quota-dependent functions on the nutrient uptake rates used in the model.**

***Starch and lipid degradation rates***

The starch and lipid degradation rates (equations 13 and 14 in the main text) incorporate two Contois-type saturating functions:

| $R_{2}=r_{2}\cdot\frac{X}{q_{N}}\cdot\frac{S/X}{S/X+k_{sat,S}}$ $R_{4}=r_{4}\cdot\frac{X}{q_{N}}\cdot\frac{L/X}{L/X+k_{sat,L}}$ | (saturated) |
| --- | --- |

The saturating functions avoid unfeasible starch and lipid accumulation scenarios which are observed when a more simple but unsaturated structure is employed:

| $R_{2}=r_{2}\cdot\frac{X}{q_{N}}$ $R_{4}=r_{4}\cdot\frac{X}{q_{N}}$ | (unsaturated) |
| --- | --- |

As observed in Figure S1, the improved “saturated” model bounds starch and lipid formation within steady concentration profiles, as opposed to the “unsaturated” model where storage molecules can attain either negative or ever-increasing concentrations.

**
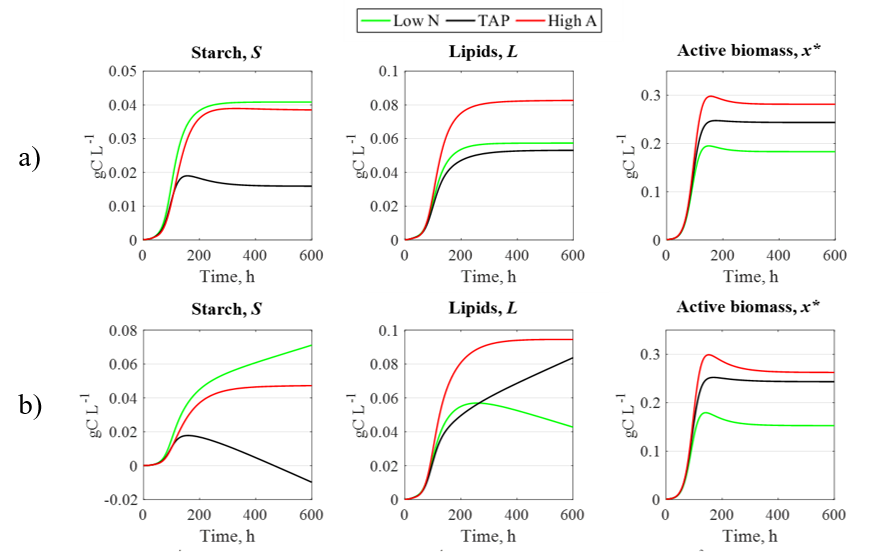
**

**Figure S3. Cultivation dynamics subject to three cultivation conditions, simulated by the a) “saturated” model, and the b) “unsaturated” model.**

1. **Model results**


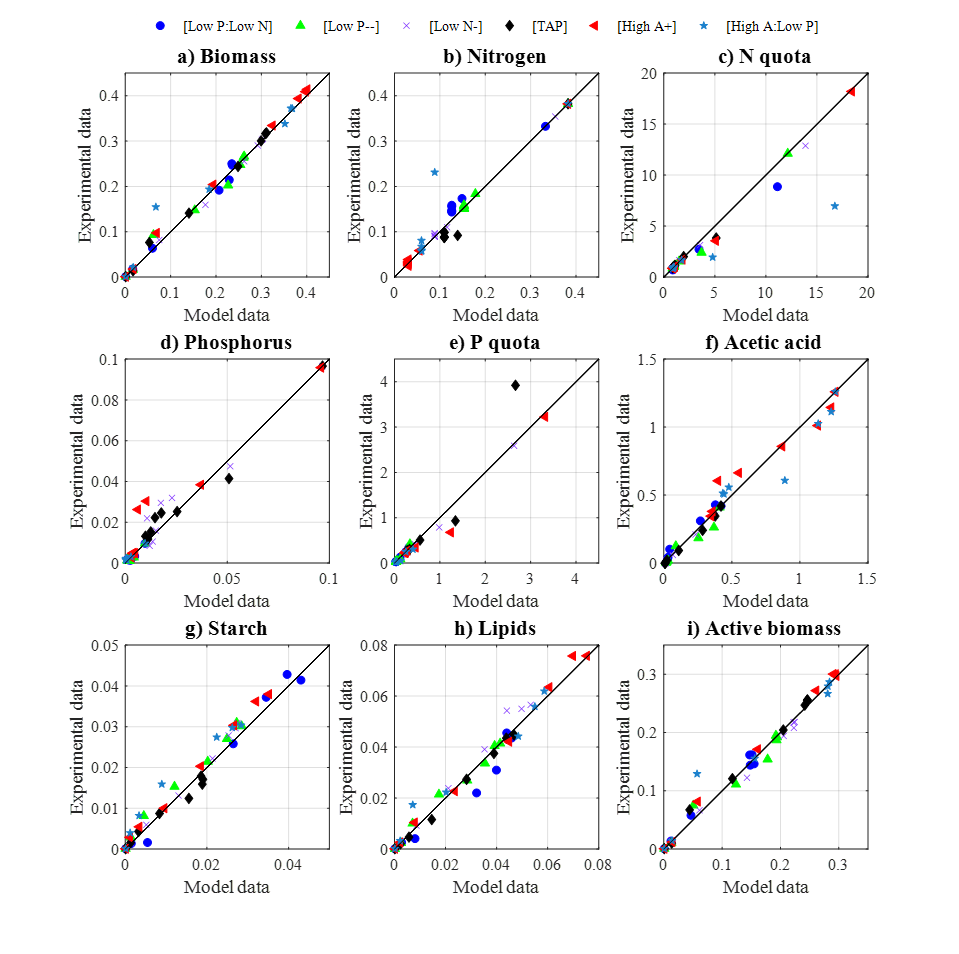


Figure S4 Parity plots comparing predicted and experimental data for both fitting and validating datasets for: a) biomass, b) nitrogen, c) nitrogen quota, d) phosphorus, e) phosphorus quota, f) acetic acid, g) starch, h) lipids, and i) active biomass. Data are the mean of two independent experimental replicates.

1. **Normalised sensitivity analysis.**

To assess the effect of the model kinetic parameters on the state variables, a normalised sensitivity analysis was performed by calculating:

$$Normalised sensitivity=\frac{\partial\bar{Z}_{i}}{\partial\bar{P}_{i}}$$

Where $\bar{Z}_{i}$=${Z_{i}}/{Z_{i,o}}$is a normalised state variable and $\bar{P}_{i}$=${P_{i}}/{P_{i,o}}$ a normalised model parameter. The normalised sensitivity, ${\partial\bar{Z_{i}}}/{\partial\bar{P}_{i}},$ denotes the normalised response change in a model state variable with respect to a corresponding normalised change in a model parameter, $Z_{i,o}$ being the response of the state variable when the parameter is set to $P_{i,o}$. To approximate the derivative through finite differences, a small 1% change value was used for computations, so that:

$$Normalised sensitivity=\frac{\partial\bar{Z}_{i}}{\partial\bar{P}_{i}}\cong\frac{\Delta\bar{Z}_{i}}{\Delta\bar{P}_{i}}=\frac{\left( Z_{i}-Z_{i,0} \right)/{Z_{i,o}}}{\left( P_{i}-P_{i,0} \right)/{P_{i,o}}}$$

The sensitivity reflects the outcome of a change in the parameter value: for sensitivities > 0, a change in the parameter increases the response of the model variable; for sensitivities < 0, a change in the parameter decreases the response of the model variable. Meanwhile, the greater the sensitivity, the greater the effect of the parameter. For computations, each parameters was changed with respect to their estimated value whilst keeping all other parameters constant (local sensitivity). The sensitivity was computed over a 200 h cultivation period

Model outputs for each evaluated parameter were generated using initial conditions equivalent to those used in [TAP] (No=0.382 gN/L, Po=0.096 gPO_4_/L, and Ao=0.42 gC/L), with the exception of the minimum P quota, $q_{P,0}$, where various phosphorous-limited initial conditions were used as the effect of this parameter only becomes significant as P concentration decreases (**Figure S3**). The results of the sensitivity analysis are presented in **Figure S4**.

**
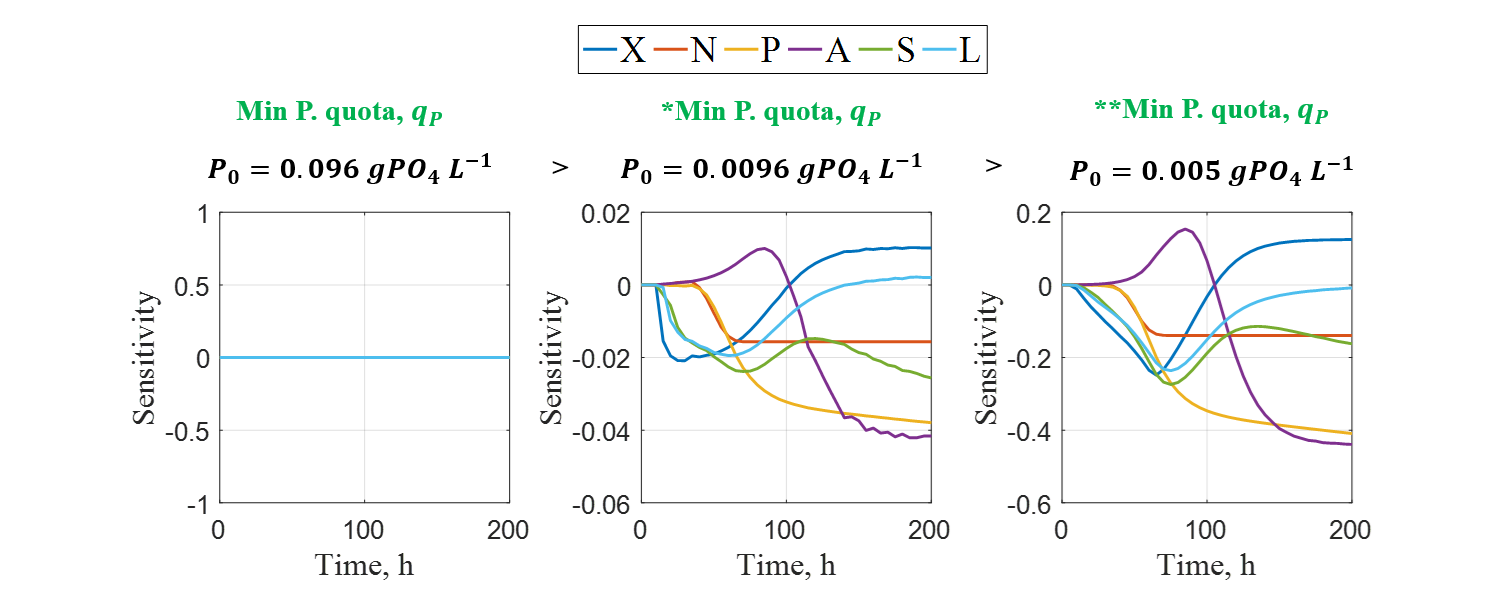
**

**Figure S5 Normalised sensitivity of the model state variables with respect to a 1 % increase in** $\boldsymbol{q}_{\boldsymbol{P,0}}$**, over a 200 h cultivation period subject to different initial phosphorous concentrations. Parameter colour denotes: green – associated to biomass growth.**

**
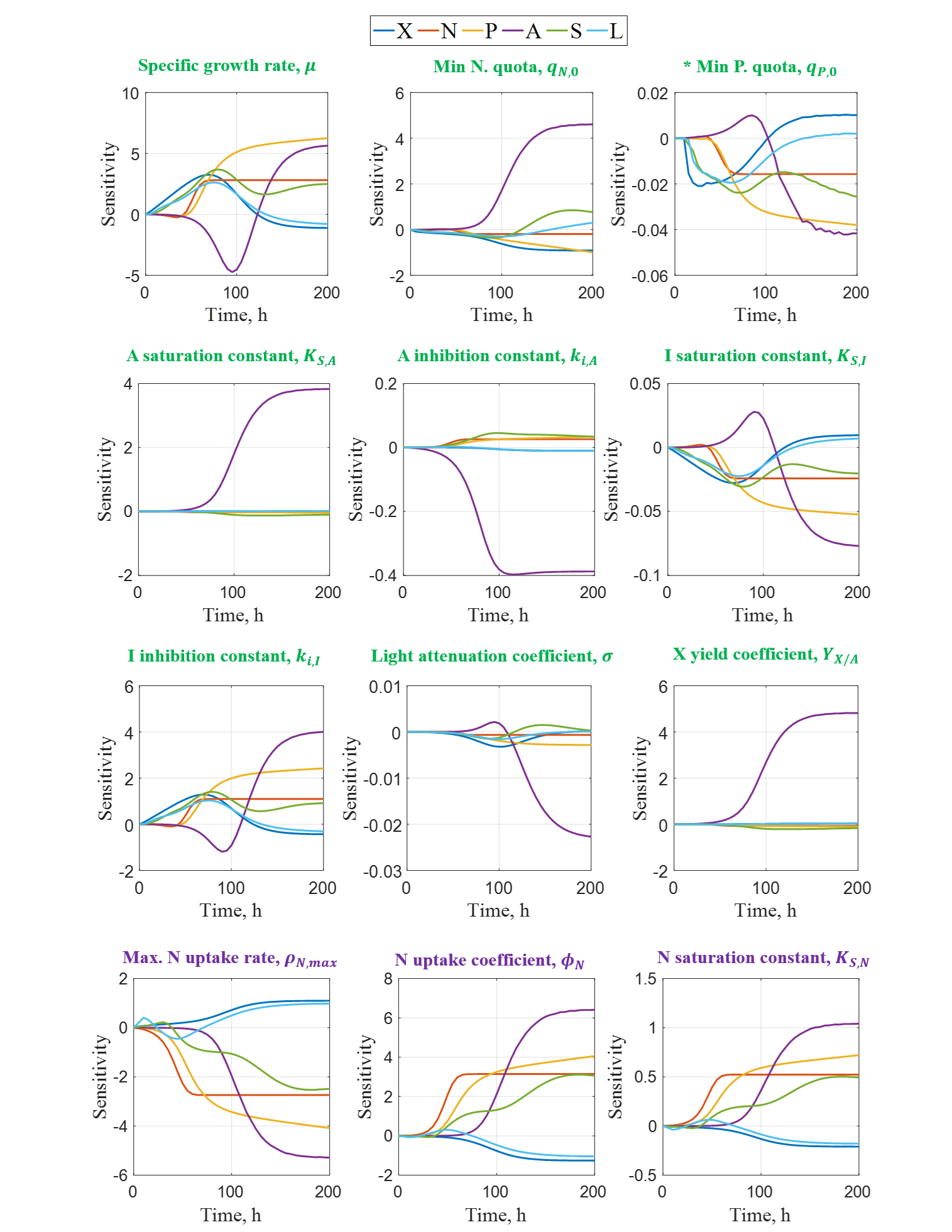
**

**Figure S6 Normalised sensitivity of the model state variables with respect to a 1 % increase in each model parameter, over a 200 h cultivation period. Parameter colours denote: green – associated to biomass growth, purple – associated to N uptake, orange – associated to P uptake, and black – associated to starch and lipid formation. * The sensitivities for** $\boldsymbol{q}_{\boldsymbol{P,0}}$ **were obtained by setting *P_0_*=0.0096 gPO4 L^-1^.**

**
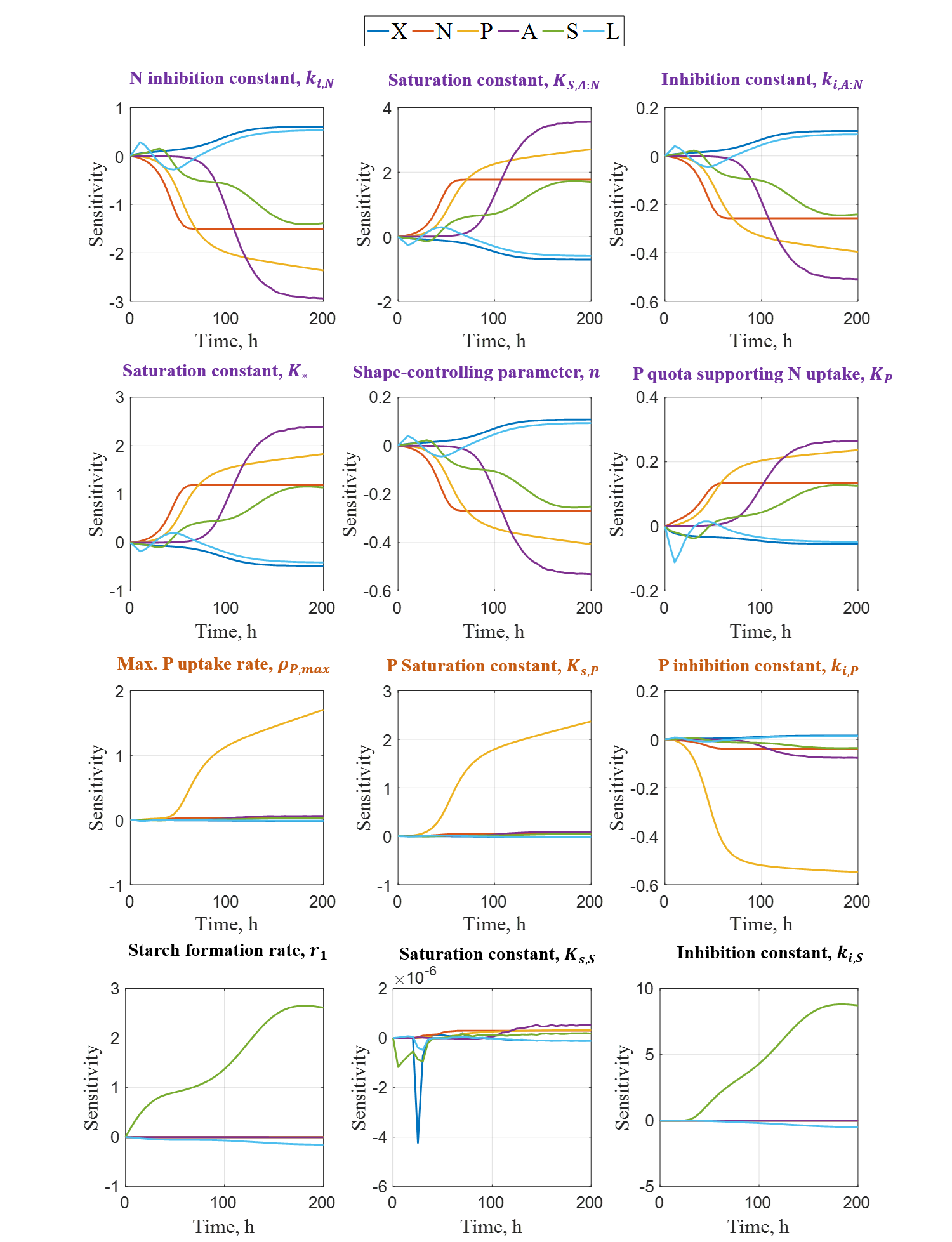
**

**Figure S6 (cont.) Normalised sensitivity of the model state variables with respect to a 1 % increase in each model parameter, over a 200 h cultivation period. Parameter colours denote: green – associated to biomass growth, purple – associated to N uptake, orange – associated to P uptake, and black – associated to starch and lipid formation.**

**
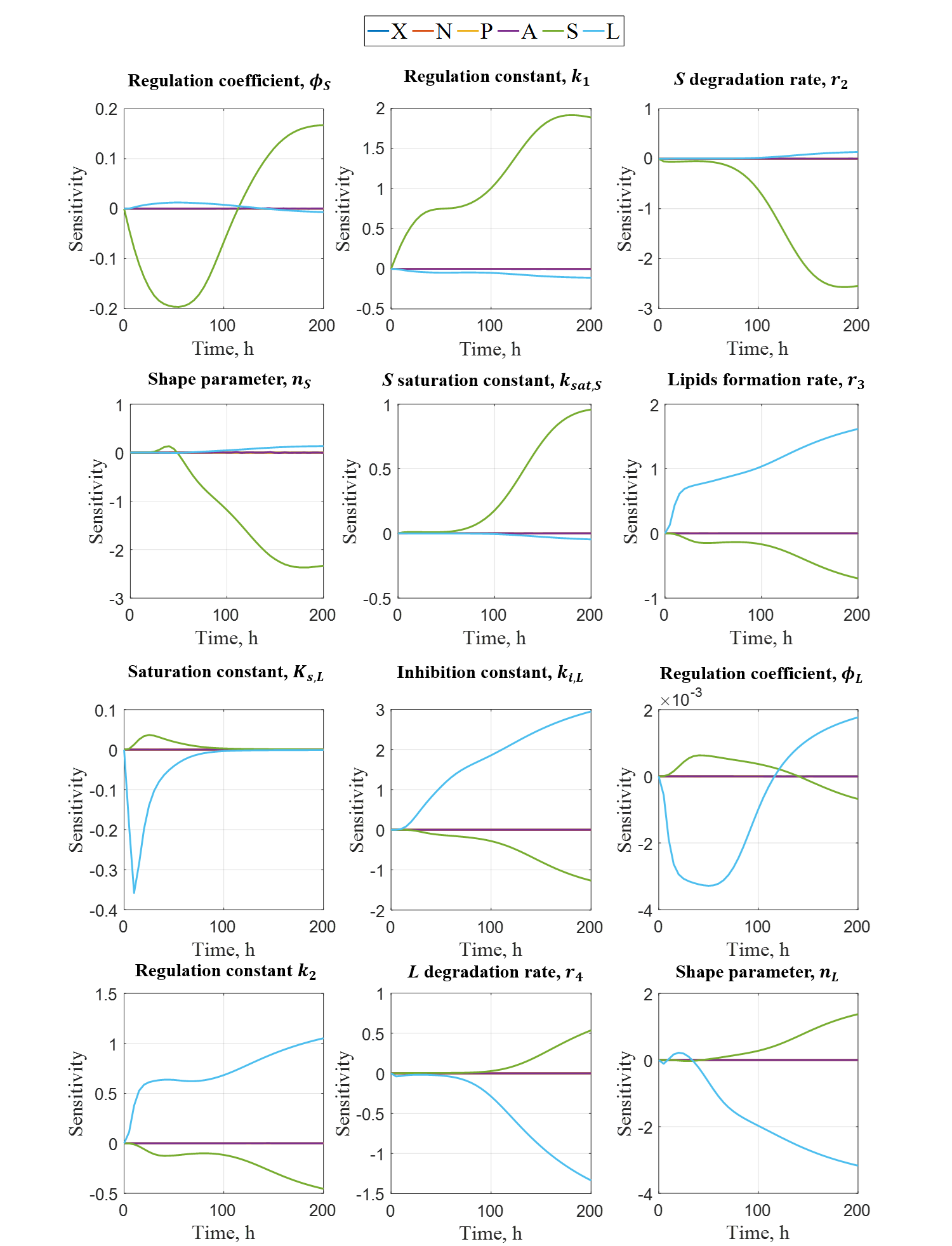
**

**Figure S6 (cont.) Normalised sensitivity of the model state variables with respect to a 1 % increase in each model parameter, over a 200 h cultivation period. Parameter colours denote: green – associated to biomass growth, purple – associated to N uptake, orange – associated to P uptake, and black – associated to starch and lipid formation.**

**
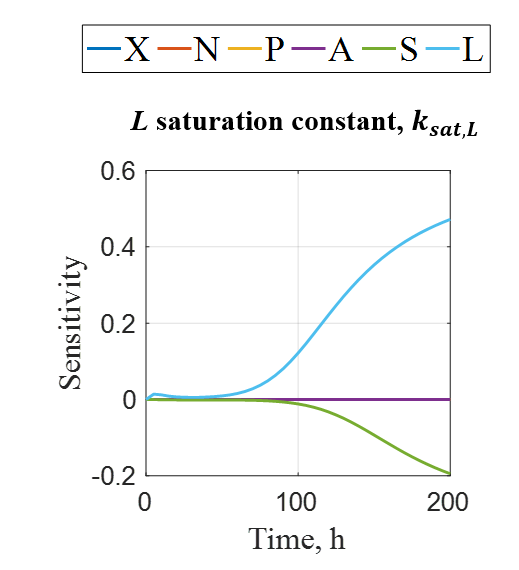
**

**Figure S6 (cont.) Normalised sensitivity of the model state variables with respect to a 1 % increase in each model parameter, over a 200 h cultivation period. Parameter colours denote: green – associated to biomass growth, purple – associated to N uptake, orange – associated to P uptake, and black – associated to starch and lipid formation.**

As per the sensitivity analysis, the specific growth rate, *μ*, is one of the most significant parameters of the model as a small change in this parameter affects state variables to a great extent (up to -5 : +5). The significance of this parameter is expected as it portrays the growth of microalgal biomass subject to the effects of the nitrogen quota, phosphorous, and acetic acid. Although the remaining parameters associated to microalgal growth (in green) have an effect on the other state variables, their effect is predominantly greater on nitrogen consumption, which regulates the nitrogen quota and, in consequence, microalgal growth.

The sensitivity analysis shown above allows to observe that the effects of the model parameters associated to biomass growth and N uptake are as significant to phosphorous as they are for nitrogen. However, whilst nitrogen-associated parameters (in purple) have an effect on all model variables, the three phosphorus-associated parameters (i.e. $\rho_{P,max}$, $K_{S,P}$ and $k_{i,P}$) (in orange) only affect phosphorous since their computed sensitivities are considerably low for the remaining variables. With respect to the parameters associated to starch and lipid formation (in black), the computed sensitivity indicates that the effect of these parameters is only significant to starch, *S*, or lipid, *L*, accumulation, respectively. The effect of these parameters on the remaining variables is negligible (sensitivity=0) given that the equations governing *X*, *N*, *P*, and *A* are completely independent of storage molecule accumulation.

As per the sensitivity analysis shown above, 4 model parameters were deemed not sensitive: $\sigma$, $k_{S,I}$, $K_{s,S}$, and $\phi_{L}$. The values of these parameters were thus set to: $\sigma=1$ (nominal value), $k_{S,I}=1.4$ (Mairet et al., 2011), and $K_{s,S}=0$ (negligible effect), and $\phi_{L}=0$ (negligible effect).
